# Generation of chimeric forms of rhesus macaque rhadinovirus expressing KSHV envelope glycoproteins gH and gL for utilization in an NHP model of infection

**DOI:** 10.1128/jvi.01923-24

**Published:** 2025-01-21

**Authors:** Ryan D. Estep, Helen Li, Aparna N. Govindan, Kaidlyn A. McDonald, Michael K. Axthelm, Scott W. Wong

**Affiliations:** 1 Vaccine and Gene Therapy Institute, Oregon Health & Science University 56870 , Beaverton, Oregon, USA; 2 Division of Pathobiology and Immunology, Oregon National Primate Research Center 88960 , Beaverton, Oregon, USA; 3 Department of Molecular Microbiology and Immunology, Oregon Health & Science University 547642 , Portland, Oregon, USA; Lerner Research Institute, Cleveland Clinic, Cleveland, Ohio, USA

**Keywords:** Kaposi's sarcoma-associated herpesvirus, glycoproteins, rhesus rhadinovirus, chimeric virus, RRV BAC, vaccines

## Abstract

Kaposi’s sarcoma-associated herpesvirus (KSHV) is a human gammaherpesvirus associated with Kaposi’s sarcoma and B cell malignancies. Like all herpesviruses, KSHV contains conserved envelope glycoproteins (gps) involved in virus binding, entry, assembly, and release from infected cells, which are also targets of the immune response. Due to the lack of a reproducible animal model of KSHV infection, the precise functions of the KSHV gps during infection *in vivo* are not completely known. Fortunately, a nonhuman primate (NHP) model of KSHV infection and disease has been established utilizing closely related rhesus macaque rhadinovirus (RRV) that naturally infects rhesus macaques (RM) and possesses analogous gps to KSHV. To address the roles conserved envelope gps gH and gL play during KSHV infection *in vivo*, we utilized the pathogenic RRV_17577_ BAC to generate chimeric forms of RRV expressing KSHV gL or KSHV gH/gL, as well as an RRV mutant lacking gL expression. These viruses incorporate KSHV gH and gL into infectious virions, and although they display variable replication and differing plaque phenotypes in primary rhesus fibroblasts, they retain the ability to infect human B cells *in vitro*. Importantly, we also demonstrate that RRV gp chimeras can infect RM and induce the development of antibodies against KSHV. Overall, this work demonstrates that RRV gp chimeras can serve as important tools to assess the role of KSHV gH/gL in infection and disease while also providing an NHP model for testing the efficacy of KSHV gH and gL neutralizing antibodies and vaccine strategies to prevent and treat KSHV infection.

**IMPORTANCE:**

Rhesus macaque rhadinovirus (RRV) is a rhesus macaque homolog of KSHV and serves as a model system for examining Kaposi’s sarcoma-associated herpesvirus (KSHV) infection and pathogenesis *in vivo*. KSHV and RRV both encode conserved herpesvirus envelope glycoproteins, including gH and gL, that are important for regulating entry into host cells. In this study, we utilized the RRV BAC system to generate chimeric forms of RRV expressing KSHV gH and gL, as well as a mutant form of RRV lacking gL expression. Although these mutant and chimeric viruses can replicate *in vitro*, they do display growth properties different from wild-type RRV. Importantly, we demonstrate that RRV gp chimeras are capable of infecting rhesus macaques *in vivo*, inducing B cell hyperplasia, and promoting the development of anti-viral antibody responses that can also recognize KSHV antigens. RRV gp chimeras provide a novel system that allows for the examination of the role of KSHV gH and gL during infection *in vivo*.

## INTRODUCTION

Kaposi’s sarcoma-associated herpesvirus (KSHV)/human herpesvirus 8 (HHV-8) is an oncogenic gammaherpesvirus that infects humans and is associated with the development of Kaposi’s sarcoma (KS), as well as B cell lymphoproliferative disorders (LPD) primary effusion lymphoma and multi-centric Castleman’s disease, and some non-Hodgkin’s lymphomas, diseases which most frequently develop in individuals co-infected with HIV (1–4). Although KSHV infection is more common in Africa, where KS can occur as an endemic disease, KSHV is also associated with classic KS in Mediterranean regions, iatrogenic KS in post-transplant recipients, as well as epidemic KS in individuals with HIV/AIDS, where it remains one of the most common AIDS-associated cancers (5). Although KS commonly affects the skin, mucosal locations such as the oral cavity can also develop lesions, with oral cavity involvement being most common in patients with AIDS-associated KS (6). Overall, KSHV displays a disease burden on a variety of populations worldwide, but despite this, there is still no available vaccine that can be utilized to prevent KSHV infection. A major hurdle to this is the fact that currently, no animal model exists that allows for reproducible KSHV infection and disease development that can be utilized to gain an understanding of the mechanisms of KSHV infection, pathogenesis, and host immune responses against the virus *in vivo* (7–9). In addition, there is also no *in vitro* system for KSHV lytic replication available, limiting the ability to generate high titer stocks of KSHV for *in vivo* studies and hampering studies of *de novo* lytic KSHV infection.

Rhesus macaque rhadinovirus (RRV) is a gammaherpesvirus that naturally infects rhesus macaques (RM) and is closely related to KSHV (10, 11). Two strains of RRV were originally identified independently in RM in the late 1990s, strains 17577 (RRV_17577_) and H26-95, with only RRV_17577_ demonstrating pathogenesis *in vivo* (12, 13). Unlike KSHV, RRV displays lytic replication in culture and can be grown to high titers. Through infection studies in RM, RRV has been shown to infect and establish a latent infection in B cells *in vivo* and induce pathologies in SIV co-infected RM that are similar to those found in KSHV/HIV co-infected humans, including B cell LPD, non-Hodgkin’s lymphomas, and a mesenchymal proliferative lesion termed retroperitoneal fibromatosis that possesses histopathological features resembling KS (12, 14, 15). Importantly, RRV is genetically similar to KSHV and encodes homologs of a variety of KSHV genes and factors hypothesized to be involved in viral pathogenesis, such as vIL-6, vGPCR, vCD200, vIRFs, and viral miRNAs, among others (10). Due to their genetic similarity and association with similar pathologies, RRV_17577_ infection of RM has proven to be a highly relevant and functional nonhuman primate (NHP) model that allows for the study of determinants of KSHV pathogenesis *in vivo* (12, 15–20).

Besides accessory genes that may affect pathogenesis, RRV also contains a wide variety of genes that are conserved throughout the *Herpesviridae* family and play critical roles in the life cycle of all herpesviruses, including viral envelope glycoproteins (gps) that are essential for virus binding to host cells and are involved in entry, assembly, and release from infected cells (10). Although the specific array of envelope gps expressed by different herpesviruses can vary between different genera and species of virus, glycoprotein B (gB), along with glycoprotein H (gH) and glycoprotein L (gL) are considered core conserved herpesvirus gps that are required for viral envelope fusion and entry into host cells (21). Given their genetic relatedness, KSHV and RRV both encode a similar array of conserved envelope gps, which include gB, gH, gL, gM, and gN, as well as the additional gp K8.1 in KSHV and its homolog R8.1 in RRV (10, 22–24). Although the role individual herpesvirus gps play in binding and entry has been highly studied in many herpesviruses such as HSV, CMV, and EBV, relatively less is known overall regarding the role of KSHV envelope gps in various stages of viral infection, particularly *in vivo*. However, the studies conducted have strongly indicated that KSHV gB and gH/gL are critical for KSHV binding and entry (25, 26). Like other conserved herpesvirus gB molecules, KSHV gB is a type I membrane protein that is incorporated into the viral envelope (25, 27). KSHV gH is also a type I membrane protein that is inserted in the envelope, while KSHV gL is a secreted protein that lacks a transmembrane domain and forms a heterodimeric complex with gH (gH/gL) through non-covalent interactions that occur with gH on the exterior of the viral envelope (28, 29). In the case of KSHV, gB is thought to be the main gp to initiate binding to most cell types, which largely occurs via nonspecific interactions with cell surface heparan sulfate proteoglycans and possibly other molecules in some cell types (22, 30, 31). After the initial virus binding, further interactions of other viral envelope gps with secondary cellular receptors are thought to regulate fusion and virus entry, which ultimately helps to determine cell tropism (30). In KSHV, gH and gL currently appear to be the most critical viral gps in terms of defining secondary interactions that regulate entry, which occur through interactions with cell surface molecules such as integrins, dendritic cell-specific intercellular adhesion molecule-3-grabbing non-integrin, xCT (cystine–glutamate antiporter), and erythropoietin-producing hepatocellular carcinoma receptors (Ephs) (22, 30). After binding with secondary cellular receptors, gH/gL is then believed to promote conformational changes in gB to induce fusogen formation, ultimately promoting envelope fusion and virus entry. These functionalities are thought to be generally conserved between KSHV and RRV gH/gL, although some variances between these molecules do appear to exist (32, 33).

Though many studies examining KSHV gH/gL expression, structure, and receptor binding have utilized proteins expressed outside the context of intact virions (28, 29), *in vitro* infections utilizing KSHV have also examined cell type dependence and receptor usage and seem to corroborate that gH and gL are critical for infection of a variety of cell types, while also confirming that secondary receptors, such as Ephs, are directly involved in interactions with KSHV gH/gL and essential for infection of most cell types examined (34–36). However, it is worth noting that the viruses used in these studies are all derived from cell lines harboring latent KSHV, and specific examination of the roles of KSHV gH/gL using a model of lytic infection and replication has not been performed. In the case of RRV, studies of the roles of RRV gps during infection are thus far more limited but have demonstrated that RRV gH/gL is critical for regulating infection of certain cell types, such as B cells and endothelial cells, through interactions with Ephs expressed on these cells, while infection of other cell types, such as fibroblasts and epithelial cells, is not completely dependent on this interaction (33, 37). In addition to Ephs, recent studies have also defined a second receptor family, the plexin domain-containing proteins 1 and 2 (Plxdc1/2), as entry receptors for RRV and targets of RRV gH/gL binding, a functionality that is not conserved in KSHV gH/gL, demonstrating the potential for gp sequence variations to affect the tropism of these different viruses (32). *In vivo* studies of RRV gH/gL function are currently limited but have demonstrated that an RRV H26-95 gL deletion mutant retains infectivity in RM, suggesting RRV gL is not specifically required for infection *in vivo* (38). However, to date, the contributions of conserved envelope gps to RRV-associated disease development have not been assessed, and the mechanisms by which they may affect viral tropism *in vivo* have not been examined. Also of importance is that significant sequence variability has been found to exist between gH and gL molecules from different isolates of RRV, with gH and gL displaying the most variability of all the conserved envelope gps. Interestingly, however, comparison of gH and gL sequences results in the clustering of each virus isolates into one of two distinct phylogenetic groupings, based solely on the similarity of gH and gL molecules to those of either RRV_17577_ or H26-95 (39), suggesting the potential for gp variation to affect a variety of aspects of infection even between two closely related viruses.

Some drawbacks often faced in studies of KSHV envelope gp function are the lack of a robust *in vitro* model of KSHV lytic replication, which hinders the ability to fully decipher the contributions of envelope gps to binding, entry, and replication during *de novo* lytic infection, and the lack of an *in vivo* model of KSHV infection that allows for the ability to study the contributions of individual gps to infection, immunity, and pathogenesis. Of particular importance, as gH/gL has been identified as the predominant target of neutralizing antibody responses in KSHV-infected humans (40), further assessing the mechanisms of gH/gL-induced immune responses during KSHV infection will be critical toward developing strategies for vaccine development that target these viral proteins, as an approach to prevent or treat KSHV infection in humans. Toward this end, we utilized the infectious and pathogenic RRV_17577_ BAC system to generate mutant and KSHV gp chimeric viruses to examine the contributions of RRV_17577_ gH/gL to infection and assess the feasibility of utilizing RRV as a model to study the function of KSHV gH and gL in the context of a highly related pathogenic virus during both *in vitro* and *in vivo* infection. Using BAC cloning, we successfully generated an RRV gL non-sense mutant (RRV gLns) lacking expression of gL and chimeric forms of RRV_17577_ expressing KSHV gL (RRV KgL) or KSHV gH and gL together (RRV KgH/KgL). Despite possessing varying replication kinetics and plaque phenotypes relative to WT RRV BAC, these mutant and chimeric viruses all demonstrate lytic replication *in vitro* and retain the ability to infect B cells, a natural target of RRV infection. Of further importance, *in vivo studies* indicate that RRV gLns, RRV KgL, and RRV KgH/KgL are all capable of infecting RM, in which they induce B cell hyperplasia and promote the development of antibody responses. The ability to generate functional chimeric forms of RRV_17577_ that express KSHV gps provides a novel approach to assessing the function of these molecules *in vivo* and can also provide new methods to examine the functionality of vaccines that target the envelope gps of KSHV.

## MATERIALS AND METHODS

### Cells

Primary rhesus fibroblasts (1^o^RF) were grown in DMEM (Corning, Manassas, VA, USA) supplemented with 10% fetal bovine serum (HyClone, Logan, UT, USA) and Penicillin-Streptomycin-Glutamine (Gibco). Human BJAB B cells were maintained in RPMI (Corning) supplemented with 10% fetal bovine serum (HyClone) and Penicillin-Streptomycin-Glutamine (Gibco).

### Construction of glycoprotein non-sense mutants and KSHV glycoprotein chimeric viruses utilizing the RRV_17577_ BAC

To generate forms of RRV lacking expression of RRV gH or gL, or chimeric forms of RRV expressing KSHV gH or gL, we utilized the RRV_17577_ BAC in conjunction with a *galK* positive/negative selection system, as described previously (16). Briefly, a clone of *Escherichia coli* strain SW105 containing the RRV_17577_ BAC was induced to express recombination genes and then electroporated with a DNA fragment containing the *galK* expression cassette flanked with RRV genomic sequence derived from either side of RRV ORF22 (gH) or RRV ORF47 (gL) start and stop codons. The primers utilized to generate these repair cassettes include 40 nt of RRV genomic sequence directly flanking either the 5′ end of the ORF22 start codon (nt 33,714 to nt 33,753) and the 3′ end of the ORF22 stop codon (nt 35,908 to nt 35,869), or the 5′ end of the ORF47 start codon (nt 65,771 to nt 65,732) and the 3′ end of ORF47 stop codon (nt 65,182 to nt 65,221), linked to sequence specific for the amplification of the *galK* cassette from the plasmid pGalK (ORF22 5′ flanking primer: 5′-CTTGAAAGCTACATTCAAACGCTAACCAAATTGGAAGGCA*CCTGTTGACAATTAATCATCGGCA* 3′, ORF22 3′ flanking primer: 5′-CGGGAAACAACCTTTTAAAGTTTTACGCTTTATTAACAGT*TCAGCACTGTCCTGCTCCTT*-3′; ORF47 5′ flanking primer: 5′-TATATAAGTACAGCATTAGCGTTCATATTATGTATAAAAC*CCTGTTGACAATTAATCATCGGCA*-3′, ORF47 3′ flanking primer sequence: 5′-GAGGGTTCTTCTAACACTTCTGGTGACATTTTACTCCAAA*TCAGCACTGTCCTGCTCCTT*-3′; RRV genomic sequence is underlined, and sequences homologous to the *galK* cassette sequence are italicized). Recombination of these PCR products with the WT RRV BAC in *E. coli* results in the precise deletion of ORF22 or ORF47 and their replacement with a *galK* expression cassette. To identify BAC clones deleted of ORF22 or ORF47 after selection, DNA was purified from *E. coli* by standard alkaline lysis methods and digested with BamHI, samples were run on a 0.7% agarose gel and transferred to a nitrocellulose membrane, and Southern blot analysis was performed using DNA probes specific to *galK*, RRV ORF22, or RRV ORF47 sequence, which were labeled with digoxigenin using a DIG-High Prime kit (Roche, Indianapolis, IN, USA). Clones identified in this fashion were then screened by PCR and sequencing across the deleted region to further confirm the correct deletion of ORF22 or ORF47.

To insert stop mutations into the RRV genome that prevent the expression of RRV gH or gL, overlapping PCR was utilized with RRV_17577_ genomic DNA as a template to generate repair fragments designed to contain stop codons in all three reading frames in the 5′ sequence of RRV ORF22 (gH) or ORF47 (gL), flanked by homology arms composed of RRV genomic sequence from either side of RRV ORF22 or ORF47, respectively, for the purposes of recombination. RRV ORF22 3× non-sense (ns) overlapping internal primers convert RRV nt 33,772 and 33,773 from AT to TA, generating a TAA stop codon in the first reading frame that is located 16 bp downstream of the ORF22 start codon and 5 bp downstream of the ORF21 stop codon, while stop codons naturally exist in the second (TAA nt 33,788–33,790) and third (TGA nt 33,822–33,824) reading frames of ORF22 (overlapping internal mutagenic primers: RRV ORF22 3× stop forward-5′ GTATTAGTTTT**TAA**TTTTTTTTTACTATAATAAGGTGTTCTGT 3′, RRV ORF22 3× stop reverse - 5′ ACAGAACACCTTATTATAGTAAAAAAAAA**TTA**AAAACTAATAC 3′; engineered stop codon is in bold, and mutated nucleotides are underlined). RRV ORF47 3× non-sense overlapping internal primers convert RRV nt 65,731 and nt 65,730 from AT to TA, generating a TAA stop codon in place of the start codon of ORF47 and also convert nt 65,721 from T to G to generate a TAG stop codon in the third reading frame, while a TAA stop codon exists naturally in the second reading frame from nt 65,712–65,710 (overlapping internal mutagenic primers: RRV ORF47 3× stop forward-5′ ATAAAAC**TAG**AGAAG**TAG**GTATACCTTAAG 3′, RRV ORF47 3× stop reverse-5′ CTTAAGGTATAC**CTA**CTTCT**CTA**GTTTTAT 3′; engineered stop codons are in bold, and mutated nucleotides are underlined). The flanking primers used in conjunction with overlapping internal primers contain 250 bp of RRV sequence directly upstream or downstream of RRV ORF22 or ORF47 and restriction sites for cloning (upstream RRV ORF22 primer + BamHI: 5′ TCC*GGATCC*CCCATGGTGGCAGAAATGTT 3′, downstream RRV ORF22 primer + ClaI: 5′ TGGT*ATCGAT*CCCACGACTTTCAGAAGGAT 3′; upstream RRV ORF47 primer + EcoRI: 5′ TGCCA*GAATTC*ATTTTATTAACATGGTGTTA 3′, downstream RRV ORF47 + XhoI 5′ AATT*CTCGAG*TGGTAAGGATCCTGGCCAAG 3′; RRV sequence is underlined, and restriction sites are in italics).

To generate chimeric forms of RRV BAC that express KSHV gH or gL, repair fragments were designed to amplify fusion products by overlapping PCR using KSHV BAC16 DNA and RRV_17577_ genomic DNA as templates. For a KSHV ORF22 (gH) chimeric, a repair fragment sequence encoding a hybrid RRV gH/KSHV gH protein was generated by using overlapping primers that generate products that ultimately fuse RRV nt 33,798 (RRV ORF22 nt 45) with KSHV nt 36,976 (KSHV ORF22 nt 46) (R22nt45-K22nt46 forward-5′ TTTTTTACTATAATAAGGTGT*ATATCGTTGACATGTGGAGCC* 3′, R22nt45-K22nt46 reverse-5′ *GGCTCCACATGTCAACGATAT*ACACCTTATTATAGTAAAAAA 3′; RRV sequence is underlined, and KSHV sequence is italicized). These primers were used in PCR in conjunction with an additional overlapping primer pair designed to place the stop codon of KSHV ORF22 outside the 3′ coding sequence of RRV ORF23 (KSHV ORF22 nt 39,120-RRV ORF23 nt 35,866 forward-5′ *GACTGTTTTCCATCCTTTAT*TAAACTGTTAATAAAGCGTA 3′, RRV ORF23 nt 35,866-KSHV ORF22 nt 39,120 reverse-5′ TACGCTTTATTAACAGTTTA*ATAAAGGATGGAAAACAGTC* 3′; RRV sequence is underlined, and KSHV sequence is italicized) and the 250 bp RRV ORF22 flanking primers described above. For KSHV ORF47 (gL), a repair fragment encoding KSHV ORF47 flanked by RRV genomic sequence outside ORF47 was generated using an overlapping primer set that inserts the stop codon of KSHV ORF47 just outside the 5′ sequence containing the start codon of RRV ORF46 (nt 65,244–65,246) in the RRV genome (RRV nt 65,249-KSHV nt 69,228 forward - 5′ TTAAGCCAACCCTC**CAT**AAA***TTA****TTTTCCCTTTTGACCTG* 3′, KSHV nt 69,228-RRV nt 65,249 reverse-5′ *CAGGTCAAAAGGGAAAA**TAA***TTT**ATG**GAGGGTTGGCTTAA 3′; RRV sequence is underlined, KSHV sequence is in italics, and start and stop codons are in bold) and were used in conjunction with another overlapping primer set that inserts the start codon of KSHV ORF47 precisely in place of the start codon for RRV ORF47 (nt 65,729–65,731) in the RRV genome (KSHV nt 69,731-RRV nt 65,754 forward-5′ *AATAGCGCAAAGATCCC**CAT***GTTTTATACATAATATGAAC 3′, RRV 65,754-KSHV nt 69,731 forward - 5′ GTTCATATTATGTATAAAAC***ATG****GGGATCTTTGCGCTATT* 3′ RRV; RRV sequence is underlined, KSHV sequence is in italics, and start codon is in bold). For each specific repair fragment, resulting overlapping PCR products were pooled and utilized in a final PCR using the 250 bp ORF22 or ORF47 flanking primers described above, generating a single product that was then digested with the indicated restriction enzymes and cloned into plasmid pSP73 (Promega, Madison, WI, USA). The resulting clones containing the repair fragment inserts were analyzed by restriction digest analysis and fully sequenced to confirm their identity.

To generate RRV_17577_ BAC clones containing designed ns mutations or expressing KSHV gps, cloned repair fragments were isolated from pSP73 by restriction enzyme digestion, gel purified, and used to electroporate competent SW105 *E. coli* containing either ORF47 or ORF22 *galK* KO RRV BAC clones that had been induced to express recombination genes. After selection, DNA was purified from *E. coli* by standard alkaline lysis methods and digested with BamHI, samples were run on a 0.7% agarose gel, transferred to a nitrocellulose membrane, and Southern blot analysis was performed using DNA probes specific to repair fragment sequences that were labeled with digoxigenin using a DIG-High Prime kit (Roche). To further confirm the correct insertion of the repair cassettes, PCR was performed to amplify across the repaired region with primers that bind outside of the recombination arms, and the resulting products were subjected to DNA sequencing for validation.

Once confirmed, the resulting RRV ORF22 and ORF47 ns mutant and KSHV gp chimeric RRV BAC clones were utilized to generate infectious virus following methods described previously (18). Briefly, purified BAC DNA was transfected into 1^o^RF, and virus produced by these cells was collected and passed twice through 1^o^RF transfected with a plasmid expressing CRE recombinase to allow the removal of the BAC cassette via recombination of *loxP* sites located within the cassette sequence. The resulting virus was then utilized in a plaque assay on 1^o^RF, and BAC-derived virus isolates lacking the BAC cassette were identified via PCR screening and DNA sequencing of the PCR products. Stocks of each virus were then grown in 1^o^RF and purified twice by ultracentrifugation through a 30% sorbitol cushion. Titers of viral stocks were assessed by plaque assay on 1^o^RF using methylcellulose overlay and were fixed with methanol and stained with crystal violet or neutral red for visualization. WT RRV BAC used in these studies was generated similarly and has been described previously (18). Growth curves were performed with purified viral stocks in 1^o^RF, and levels of infectious virus in samples were measured by plaque assay on 1^o^RF using methylcellulose overlay, followed by fixation with methanol and staining with crystal violet for visualization of plaques.

Confirmation of the DNA sequence and integrity of the entire genome of each virus was performed by subjecting viral genomic DNA to sequencing with an Illumina iSeq (Illumina, Inc., San Diego, CA, USA). Complete viral genome sequences were submitted to GenBank (RRV gLns accession number PP101847, RRV KgL accession number PP101848, and RRV KgH/KgL accession number PP101849). All nucleotide numbers listed in the text are in reference to the genomic sequence of RRV_17577_ (GenBank accession number AF083501.3).

### RT-PCR analysis of viral gene expression

To assess transcriptional expression patterns of gp ORFs and neighboring genes in ns mutant and chimeric viruses, 1^o^RF were infected with WT RRV BAC, RRV gLns, RRV KgL, or RRV KgH/KgL at an MOI of 2, and RNA was isolated from infected cells 72 hours post-infection (pi) using a Direct-zol RNA Miniprep Plus Kit (Zymo Research, Irvine, CA, USA). RT-PCR analysis was performed with 100 ng of purified RNA using a Qiagen One-step RT-PCR Kit (Qiagen, Germantown, MD, USA). Primers used include those specific for RRV ORF22 (R22 forward: 5′ AGCAAAGCCCGATGACATCA 3′, R22 reverse: 5′ TGGCTTGAACTGTGGGGTTT 3′), KSHV ORF22 (K22 forward: 5′ CCACGAAGTTGGCCAGTTTG 3′, K22 reverse: 5′ TAAGCTATCGAGGTGCAGCG 3′), RRV ORF21 (R21 forward: 5′ GACCCTCGTCGACAAAACCT 3′, R21 reverse: 5′ AACTTGCTCTGACACGCGTA 3′), RRV ORF23 (R23 forward: 5′ GCCCGATTACCGCGTTTAAC 3′, R23 reverse: 5′ ATTCAATCACCTCTGCGCCA 3′), RRV ORF47 (R47 forward: 5′ ACATGTTGTACCGGGCTTGT 3′, R47 reverse: 5′ AGCTCTTCTCTGGGGACACT 3′), KSHV ORF47 (K47 forward: 5′ TACCGTGGAAACATGCGTGA 3′, K47 reverse: 5′ TGGCCTTTCCCACGCTTATT 3′), RRV ORF46 (R46 forward: 5′ TGGTGGTCGTATTGTTGCGA 3′, R46 reverse: 5′ CGTTTATCAGCGTCGCCTTG 3′), and RRV ORF 48 (R48 forward: 5′ AAAATTGGCACGCTCCACAC 3′, R48 reverse: 5′ GCTCGTCTCTTAACCGCAGT 3′). Reactions performed on each sample using GAPDH primers with or without RT served as controls for RNA purity.

### Analysis of glycoprotein incorporation into chimeric viruses

Western blot analysis to assess gp incorporation into purified RRV virions was performed by loading and running equivalent PFUs of gradient purified RRV stocks on a denaturing 10% acrylamide gel, then transferring the protein to a nitrocellulose membrane. Antibodies used for probing membranes include mouse anti-KSHV gH antibody 57C12 (a gift from Dr. J. Gordon Ogembo, City of Hope), rabbit anti-KSHV gL antibody UK170 (a gift from Dr. Bala Chandran), and mouse anti-RRV MCP. KSHV used in western blots was purified from the supernatants of iSLK.219 cells induced with doxycycline (41).

### Immunofluorescence analysis of KSHV gL and RRV gH localization

To examine the localization of KSHV gL and RRV_17577_ gH, immunofluorescence analysis was performed in HEK293T/17 cells. Briefly, KSHV ORF47 (gL) and KSHV ORF22 (gH) containing an in-frame C terminal HA tag were amplified by PCR from BAC16 DNA, while RRV ORF22 (gH) with an in-frame C terminal HA tag was amplified by PCR from WT RRV_17577_ BAC DNA, and the resulting products were cloned into vector pcDNA3.1(−) (Invitrogen) and sequenced to confirm their identity. Next, HEK293T/17 cells were transfected with KSHV ORF47 (gL), RRV ORF22 (gH)-HA, or KSHV ORF22 (gH)-HA expressing plasmids using TransIT-LT1 transfection reagent (Mirus Bio). Cells were also co-transfected with a plasmid encoding RRV ORF57 to enhance the expression of RRV gH (42). At 48 hours post-transfection, cells were fixed with methanol and stained using rabbit anti-KSHV gL antibody UK170 and mouse anti-HA antibody (Sigma-Aldrich), followed by anti-rabbit-FITC and anti-mouse Texas Red secondary antibodies (Vector Laboratories). DAPI staining was also performed to visualize nuclei. Images were obtained with a Zeiss Axio Imager.M1 microscope (Zeiss Imaging Solutions, Thornwood, NY, USA) equipped with a Zeiss AxioCam camera (MRm), using a 63× oil immersion lens. Images were acquired, and z-stacks were performed using ZEN 2.5 pro software (Zeiss).

### BJAB infection assays

To establish long-term infected BJAB cultures, cells were infected at an MOI of 2 in the presence of polybrene (4 μg/mL final concentration) for 2 hours. The cells were centrifuged and washed twice with PBS, resuspended in fresh media, and placed into flasks. At indicated time points, ~2 × 10^5^ cells of each culture were collected and centrifuged, supernatants were removed, and DNA was purified from cell pellets using a Wizard Genomic DNA Purification Kit (Promega).

For binding and entry assays, all infection samples were assembled on ice using cold reagents, and for each virus and condition, 1 × 10^5^ cells were infected at an MOI of 2 in the presence of polybrene (4 μg/mL final concentration). Next, samples measuring surface-bound virus only were incubated at 4°C for 2 hours, samples measuring internalized and remaining surface-bound virus were incubated at 37°C for 2 hours, while samples measuring internalized virus only were incubated at 37°C for 2 hours, washed twice with cold PBS, and then incubated with 0.25% trypsin-EDTA (Gibco) for 10 minutes at 37°C to remove remaining surface-bound virus. After the final incubations for each condition were complete, cells were centrifuged and washed twice with cold PBS, and DNA was purified using a Wizard Genomic DNA Purification Kit (Promega).

For reactivation assays, ~0.5 × 10^6^ latently infected BJAB cells were stimulated with 5 μg/mL anti-human IgM (Sigma-Aldrich) for 48 hours or treated with NaCl carrier solution as a control. Next, cells were centrifuged, supernatants were removed, and DNA was purified from cell pellets using a Wizard Genomic DNA Purification Kit (Promega).

DNA isolated from BJAB cells was subjected to real-time PCR analysis with a primer and probe set specific for RRV ORF3 (viral macrophage inflammatory protein [vMIP]) and RM glyceraldehyde 3-phosphate dehydrogenase (GAPDH) (16), and RRV genome copy numbers (vMIP) were normalized to GAPDH copy numbers to determine the relative amount of RRV genomes in each sample. All reactions were performed using an Applied Biosystems QuantStudio 3 Real-Time PCR System (Thermo Fisher Scientific, Waltham, MA).

### Experimental animal infections

RM seronegative for RRV, rhesus cytomegalovirus, SIV, simian retrovirus type D, SV40, and herpes B virus were inoculated intravenously (i.v.) with 5 × 10^6^ PFU of purified BAC-derived RRV.

Whole blood (WB) samples were collected from all animals prior to infection (day 0) and then at days 4 and 6 pi, and weekly thereafter for the duration of the study. Plasma and peripheral blood mononuclear cells (PBMCs) were isolated from WB by centrifugation over lymphocyte separation medium (Mediatech, Manassas, VA, USA). Levels of infectious virus in PBMC were assessed by co-culture analysis with 1^o^RF as described previously (18). To determine the levels of CD20+ B cells in infected animals by flow cytometry, 400 μL of WB was resuspended in wash buffer (PBS containing 0.05% sodium azide and 0.1% BSA), centrifuged for 15 minutes at 1,800 rpm, and the resulting cell pellet was resuspended in wash buffer and surface stained with antibody directed against CD20 (BD Biosciences). Samples were acquired using an LSRII instrument, and data were analyzed using FlowJo software (FlowJo LLC, Ashland, OR, USA).

To measure antibody responses in infected animals, anti-RRV IgG levels were measured in circulating plasma using a standard enzyme-linked immunosorbent assay (ELISA) (19). For these experiments, serial threefold dilutions of plasma were incubated in duplicates on RRV virus lysate-coated ELISA plates for 1 hour prior to washing, staining with detection reagents (HRP-anti-IgG), and addition of chromogen substrate to allow for the detection and quantitation of bound antibody molecules. Log-log transformation of the linear portion of the curve was then performed, and 0.15 OD units were used as the cutoff point to calculate end-point titers. Each plate included a positive control sample used to normalize the ELISA titers between assays, and a negative control sample to ensure the specificity of the assay conditions.

At the time of necropsy, tissue samples, saliva, urine, and WB were collected from each animal, and DNA was isolated using a Wizard Genomic DNA Purification Kit (Promega). RRV DNA loads were then assessed in these samples by performing qPCR with a primer and probe set specific for RRV ORF3 (vMIP), using 100 ng of template DNA for each reaction. All reactions were performed using an Applied Biosystems QuantStudio 3 Real-Time PCR System (Thermo Fisher Scientific, Waltham, MA, USA).

To assess the reactivity of antibodies from infected RM against KSHV, western blot analysis was performed with day 49 plasma samples obtained from each animal. For these assays, KSHVs purified from iSLK.219 cells induced with doxycycline or lysate samples obtained from these same cells were run on denaturing 10% acrylamide gels, transferred to nitrocellulose, and probed with dilutions of individual plasma samples using a slot blot apparatus. Titration of each plasma sample was performed to determine the optimal dilution levels for obtaining a signal without excessive background.

## RESULTS

### Construction of glycoprotein ns and KSHV glycoprotein chimeric forms of RRV utilizing the RRV BAC

For both KSHV and RRV, gps gH and gL form a heterodimer (gH/gL) that is found on the exterior surface of the viral envelope and are believed to be directly involved in regulating fusion with cellular membranes during infection by these viruses. As such, examination of both molecules in conjunction is important in helping to address their relative contributions to virus binding and infection. Currently, the role of gH and gL during KSHV infection *in vivo* is unknown, and in the case of RRV, only one study has examined the potential role of RRV gL in infectivity *in vivo* in RM, utilizing a gL deletion of the non-pathogenic RRV strain H26-95 (38). To further address the roles of RRV gH and gL in RRV infection and determine whether KSHV gH and gL can be studied in the context of RRV as a model of *in vivo* infection and disease development, we sought to generate mutant forms of RRV lacking gH or gL expression and create chimeric forms of RRV expressing KSHV gH and gL. For our work, we utilized RRV strain 17577 (RRV_17577_), as this strain has been demonstrated to display pathogenesis *in vivo*, and a model of infection and disease development in NHP has been well established (12, 14–20). Although RRV strains H26-95 and 17577 both encode gH (ORF22) and gL (ORF47) molecules with similarity to KSHV gH and gL (Fig. S1A and B), between these strains, the most variability in conserved envelope gp sequence occurs within the gH and gL molecules, with sequenced RRV genomes having been found to cluster into two distinct phylogenetic groupings based solely on comparison of gH and gL (39). Therefore, due to gp sequence differences even between highly related viruses, the possibility exists that intermixing of paired molecules like gH and gL from different gammaherpesviruses may not always be feasible.

**Fig 1:**
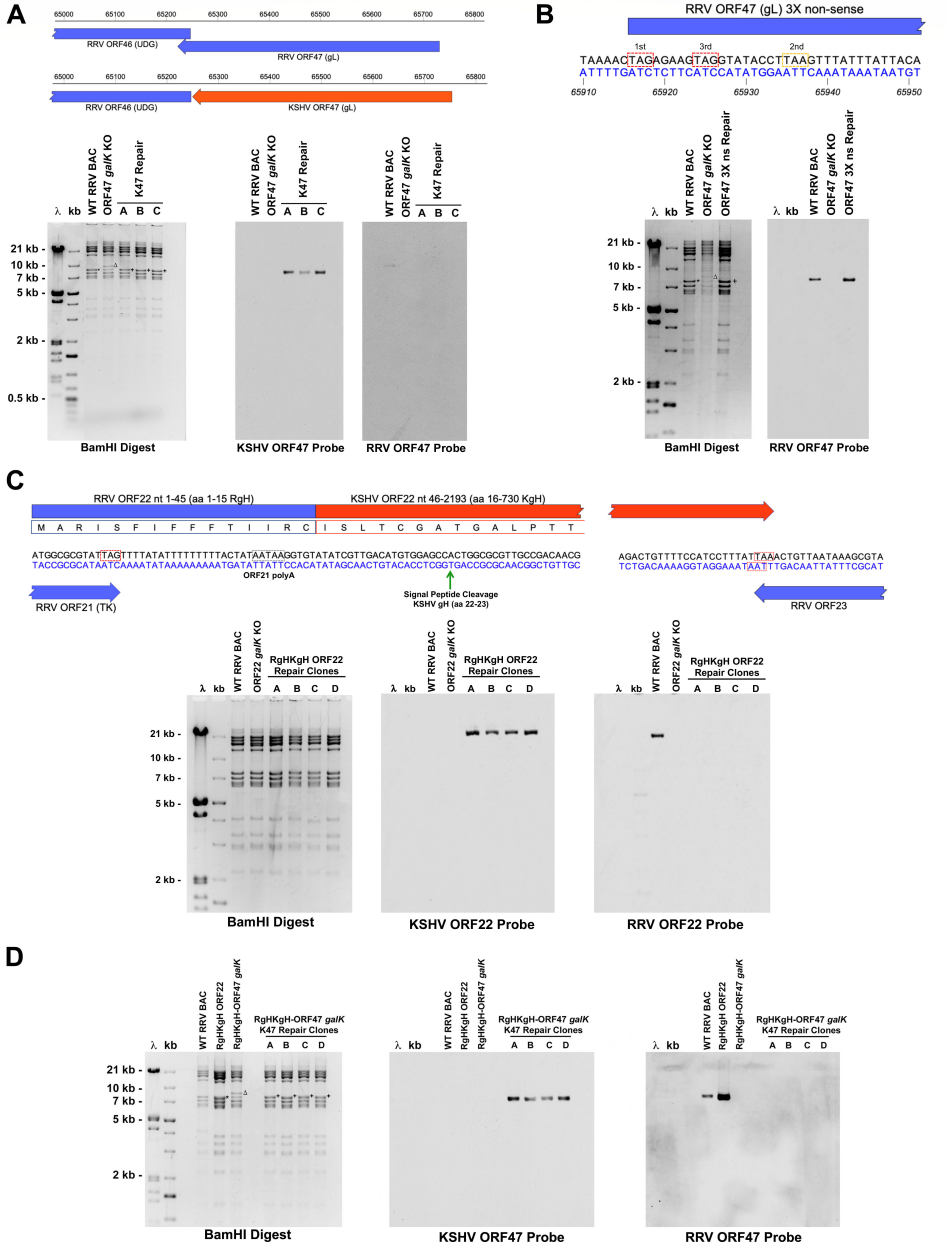
Generation of KSHV ORF47 chimeric, RRV ORF47 non-sense mutant, KSHV ORF22 hybrid chimeric, and KSHV ORF22 hybrid/ORF47 double chimeric RRV BAC clones. (**A**) Schematic depicting the insertion of KSHV ORF47 sequence in place of RRV ORF47 in the RRV genome, and BamHI restriction digestion and Southern blot analysis of RRV BAC clones. A 7.7 kb BamHI fragment in WT RRV BAC (*) shifts to 8.4 kb (Δ) upon replacement of RRV ORF47 with a *galK* cassette and reverts to 7.7 kb after repair with KSHV ORF47 sequence (+). Southern blot analysis for KSHV ORF47 was performed on digested DNA transferred to nitrocellulose, and the membrane was then stripped and reprobed for RRV ORF47. (**B**) Diagram of mutations inserted into the 5′ region of RRV ORF47 to generate an ORF47 3× non-sense repair fragment. TAG stop codons were introduced into the first and third reading frames (red dashed boxes), while a TAA stop codon in the second reading frame is naturally occurring (yellow dashed box). BamHI restriction digestion of RRV BAC clones followed by Southern blot analysis with an RRV ORF47 probe confirms the correct insertion of the ORF47 3× non-sense sequence. A 7.7 kb BamHI fragment in the wild-type RRV BAC (*) shifts to 8.4 kb (Δ) upon replacement of RRV ORF47 with a *galK* cassette and reverts to 7.7 kb after repair with ORF47 3× non-sense sequence (+). (**C**) Design of a hybrid RgHKgH ORF22 sequence encoding the first 15 amino acids of RRV gH fused to amino acids 16–730 of KSHV gH for insertion into the RRV genome. Stop codons of upstream ORF21 (TK), KSHV ORF22, and downstream ORF23 are noted by red dashed boxes, and the predicted poly A signal of ORF21 is noted by a gray dashed box. The signal peptide cleavage signal of KSHV gH is noted by a green arrow, with cleavage of the RgHKgH protein at this site allowing for the production of a processed form of KSHV gH protein lacking any RRV gH sequence. The 3′ end of KSHV ORF22 sequence was also inserted into RRV flanking arm sequence such that the stop codon of opposing ORF23 was not disrupted. BamHI restriction digestion and Southern blot analysis of RRV BAC clones confirm the correct insertion of RgHKgH hybrid ORF22 sequence in place of RRV ORF22. A 15.6 kb BamHI fragment in the wild-type RRV BAC shifts to 14.7 kb upon replacement of RRV ORF22 with a *galK* cassette and reverts to 15.7 kb after repair with RgHKgH ORF22 sequence to generate a chimeric BAC clone. Southern blot analysis for KSHV ORF22 was performed on digested DNA transferred to a nitrocellulose membrane, and the membrane was then stripped and reprobed for RRV ORF22. (**D**) Generation of a double chimeric RgHKgH hybrid ORF22/KSHV ORF47 RRV BAC clone. The same repair fragment from panel A was utilized to repair an RgHKgH ORF22 clone in which ORF47 was replaced with *galK*. BamHI restriction digestion and Southern blot analysis of RRV BAC clones confirm the correct insertion of KSHV ORF47 sequence in place of RRV ORF47 in the RRV BAC. A 7.7 kb BamHI fragment in the wild-type RRV BAC (*) shifts to 8.4 kb (Δ) upon replacement of RRV ORF47 with a *galK* cassette and reverts to 7.7 kb after repair with KSHV ORF47 sequence (+) to generate a chimeric BAC clone. Southern blot analysis for KSHV ORF47 was performed on digested DNA transferred to a nitrocellulose membrane, and the membrane was then stripped and reprobed for RRV ORF47.

Initially, we focused on generating KSHV gL chimeric and RRV gL ns forms of RRV_17577_, as RRV H26-95 lacking gL expression was previously shown to remain capable of replicating *in vitro* and maintaining infectivity *in vivo* (35, 38). To achieve this, recombination was performed to insert a *galK* cassette in place of ORF47 in the RRV BAC, followed by positive selection (Fig. S2A and B). Next, an ORF47 *galK* deleted BAC clone was used in recombination with a repair fragment containing KSHV ORF47 (gL) flanked by RRV genomic sequence. This fragment was designed to result in the insertion of the KSHV ORF47 sequence outside of downstream ORF46 (UDG), so as not to disrupt the sequence containing the start codon of ORF46. After the negative selection step, clones in which the *galK* cassette was replaced with the KSHV ORF47 sequence were identified by restriction digestion and Southern blot analysis (Fig. 1A), followed by PCR and sequencing of the inserts (data not shown). A similar approach was taken to generate a gL non-sense RRV BAC clone, by repairing an ORF47 *galK*-deleted RRV BAC clone with a mutated fragment (ORF47 3× non-sense) in which a stop codon replaces the start codon of RRV ORF47, and stop codons are also present in the second and third reading frames in the 5′ region of ORF47 (Fig. 1B). After identification of clones containing the engineered mutations or KSHV ORF47 sequence, purified BAC DNA was isolated from *E. coli* and transfected into 1^o^RF to generate infectious virus. After transfection, BAC clones containing RRV ORF47 stop codon mutations or KSHV ORF47 sequence both produced virus, which appeared after approximately 3–4 weeks in culture. These viruses were named RRV gL non-sense (RRV gLns) and RRV KgL, respectively.

Using a similar approach, attempts were made to generate RRV gH non-sense and KSHV gH chimeric viruses using the RRV BAC. Specifically, an RRV BAC clone in which ORF22 is replaced with a *galK* cassette was repaired using either an RRV ORF22 fragment containing stop codons in all three reading frames in the 5′ region of ORF22 or KSHV ORF22 sequence encoding full-length KSHV gH (Fig. S2C and D). For the RRV ORF22 stop mutant, due to overlap of the 5′ end of RRV ORF22 with the 3′ end of upstream ORF21, a TAA stop codon was inserted in the first reading frame from nt 33,772–33,774, 5 bp downstream of the ORF21 stop codon and 16 bp downstream of the ORF22 start codon, while the second and third reading frames both contain natural stop codons in this region. In the case of the KSHV ORF22 repair fragment, the inserted sequence was designed such that the start codon of KSHV ORF22 is located downstream of the stop codon of upstream RRV ORF21, and the 3′ end of KSHV ORF22 does not overlap with downstream RRV ORF23. After identification and confirmation of RRV BAC clones containing the desired sequences, it was found that neither RRV ORF22 non-sense nor KSHV gH chimeric BAC clones demonstrated any signs of virus production after transfection into 1^o^RF, even after multiple attempts (data not shown). These findings appear to agree with studies of a gH-null mutant form of KSHV, in which gH expression was found to be required for KSHV infection and replication in permissive cell types *in vitro* (43) and suggests the possible lack of expression of KSHV gH from the designed KSHV gH chimeric BAC clone, which may render this virus incapable of replication in culture. Reexamination of the 5′ region RRV ORF22 identified a potential complication in the design of this KSHV ORF22 containing BAC clone being the removal of putative regulatory sequences for the neighboring upstream ORF21 (Thymidine Kinase), including a partial polyA site in the 5′ region of RRV ORF22 located from nt 33,789 to 33,793 in the RRV genome. Therefore, the KSHV ORF22 chimeric BAC was redesigned to retain more RRV genomic sequence surrounding the stop codon of ORF21. To achieve this, a second repair construct was generated that contains 45 bp of the 5′ sequence of RRV ORF22, ultimately creating a hybrid form of ORF22 that encodes an RgHKgH fusion protein in which the first 15 aa of RRV gH is linked in frame to aa 16 of KgH, just prior to the predicted signal peptide cleavage site of KSHV gH that is located between aa 22 and 23, ultimately allowing for the production and processing of a functional form of KSHV gH from this virus that lacks any RRV sequence (Fig. 1C). As in the previous repair construct, the stop codon of KSHV ORF22 was also inserted such that the 3′ end of the opposing downstream RRV ORF23 is not disrupted. Introduction of this repair fragment in the place of RRV ORF22 *galK* resulted in the successful generation of RRV BAC clones containing RgHKgH hybrid ORF22 coding sequence (Fig. 1C). Importantly, this modified design rescues the ability of BAC clones lacking RRV gH expression to produce virus, as 1^o^RF transfected with RgHKgH hybrid chimeric BAC DNA developed cytopathic effect (CPE) in culture. However, despite this, virus generated from RgHKgH hybrid chimeric BAC clones spreads much less efficiently and appears to grow at a significantly slower rate than what is observed with WT RRV BAC DNA transfection (data not shown). Furthermore, although this virus was capable of being passaged in culture to new 1^o^RF, due to severely stunted growth, a purified plaque isolate capable of sufficient replication to generate a stock was never obtained (Fig. S1C). Overall, this may indicate that KSHV gH requires pairing with KSHV gL to remain fully functional, at least in the context of RRV infection.

Next, to promote the growth of a form of RRV expressing KSHV gH that can also be successfully propagated and purified, we generated a double chimeric form of RRV that simultaneously expresses KSHV gL as well as the hybrid RgHKgH molecule. To achieve this, the RgHKgH ORF22 chimeric RRV BAC clone was used as a target to replace RRV ORF47 with a *galK* cassette, and the KSHV ORF47 repair fragment was then utilized to repair this deletion (Fig. 1D; Fig. S2). After screening and successful identification of RgHKgH ORF22-KSHV ORF47 double chimeric RRV BAC clones, BAC DNA was transfected into 1^o^RF, which resulted in the successful and efficient production of infectious virus by ~4 weeks post-transfection. This virus, termed RRV KgH/KgL, was produced more readily than virus generated from RgHKgH chimeric BAC clones. Furthermore, it was propagated and passaged through CRE expressing 1^o^RF, allowing for the successful identification of a viral isolate lacking the BAC cassette and the growth of a high titer viral stock.

**Fig 2:**
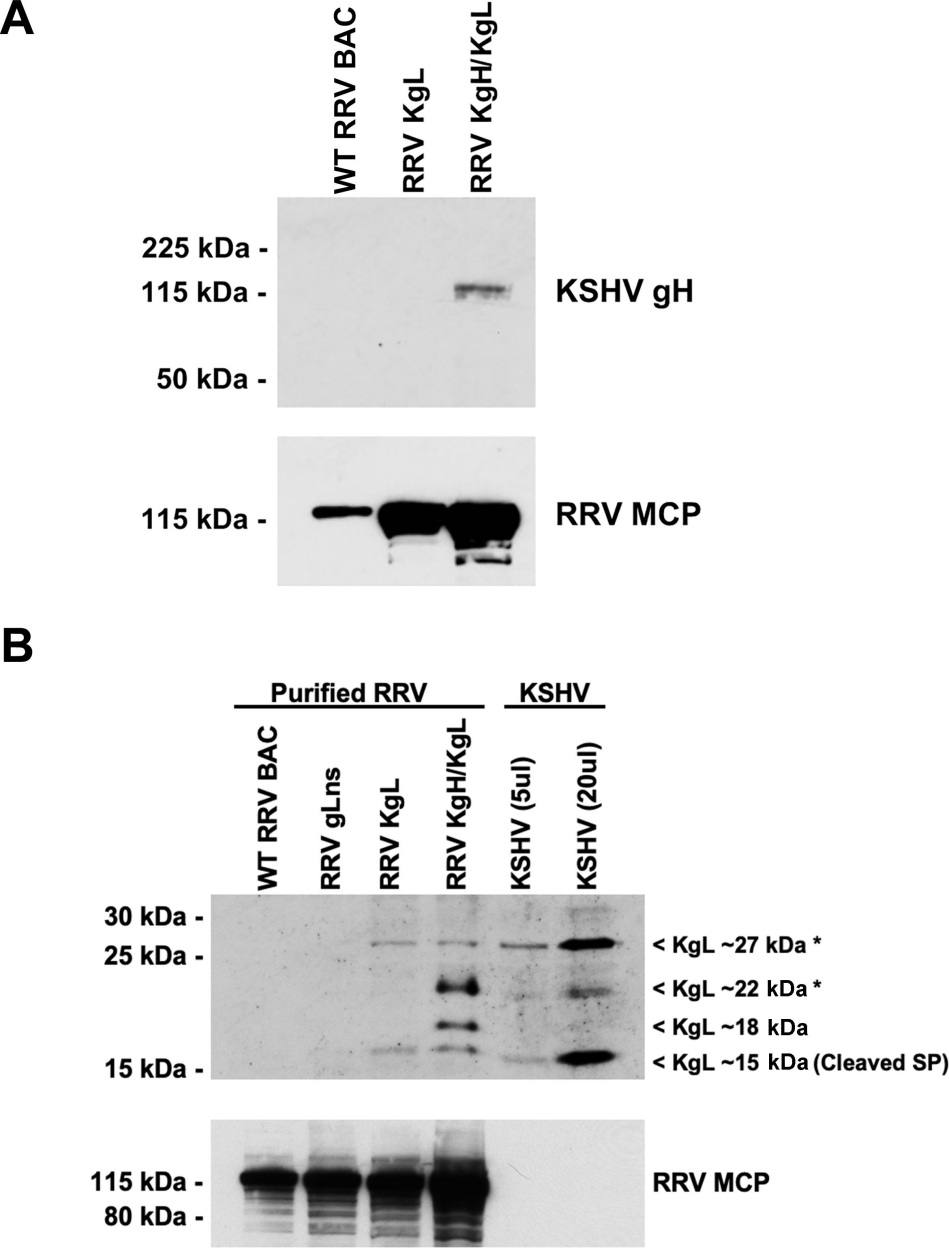
Western blot analysis of purified RRV glycoprotein chimeric virions for the incorporation of KSHV gH or KSHV gL. (**A**) Equivalent PFUs of purified WT RRV BAC, RRV KgL, and RRV KgH/KgL were loaded and run on a 10% acrylamide gel, transferred to a nitrocellulose membrane, and probed with antibody specific for KSHV gH. The membrane was then stripped and re-probed for RRV major capsid protein (MCP) as a loading control. Signal for KSHV gH is only detected in RRV KgH/KgL virions. (**B**) Equivalent PFUs of purified WT RRV BAC, RRV gLns, RRV KgL, and RRV KgH/KgL were loaded and run on a 10% acrylamide gel, transferred to a nitrocellulose membrane, and probed with antibody specific for KSHV gL. The membrane was then stripped and re-probed for RRV MCP as a control. A volume of 5 and 20 mL of KSHV was also loaded as a control. Common bands of ~27 and ~15 kDa are detected in all viruses expressing KSHV gL, while an ~22 kDa band is detected only in RRV KgH/KgL and KSHV virions, and an ~18 kDa band is detected specifically in RRV KgH/KgL virions. The ~15 kDa band directly correlates with the predicted size of an unmodified form of KSHV gL lacking the signal peptide sequence (cleaved SP), while the larger ~22 and ~27 kDa bands represent putative modified forms of KSHV gL (denoted by *). No signal for KSHV gL was detected in WT RRV BAC or RRV gLns virions.

Ultimately, purified stocks of RRV gLns, RRV KgL, and RRV KgH/KgL were successfully generated (Fig. S1B). After gradient purification of each virus, virion DNA was isolated and subjected to whole-genome sequencing. This analysis confirmed the correct insertion of engineered mutations and KSHV gp sequences, and the integrity of the remainder of the genomic sequence in each virus. Complete genomic sequences of each virus were submitted to GenBank. RT-PCR analysis was also performed with each virus, demonstrating the expression of transcripts from all inserted modified ORFs and confirming that the engineered alterations in RRV genomic sequence do not disrupt the transcription of RRV genes flanking ORF21 or ORF47 in any virus (Fig. S3).

### Glycoprotein expression and incorporation into RRV chimeras

To validate gp expression and incorporation into chimeric forms of RRV, virus preparations were purified by gradient ultracentrifugation, and purified virions were denatured and resolved on SDS-PAGE for western blot analysis. For these experiments, equivalent PFUs of purified virus stocks were loaded in each lane, and membranes were probed with antibodies specific for KSHV gH or KSHV gL. Utilizing a KSHV gH-specific antibody, an ~120 kDa band is readily detectable in RRV KgH/KgL double chimeric virions, while no KSHV gH signal is present in WT RRV BAC or RRV KgL virions (Fig. 2A), demonstrating that KSHV gH is expressed by and incorporated into RRV KgH/KgL. The size of KSHV gH detected in RRV KgH/KgL virions correlates with that of a glycosylated form of this molecule that has been reported by others (28, 40).

Western blot analysis of purified RRV KgL and RRV KgH/KgL virions using a KSHV gL-specific antibody reveals that both viruses express and incorporate KSHV gL, though differing sizes of this protein are detected between these viruses. Specifically, a KSHV gL-specific signal is detected at ~27 kDa in purified RRV KgL, RRV KgH/KgL, and KSHV virions (Fig. 2B), which correlates with the size of a modified form of KSHV gL that has been observed by others (28, 40, 44). In addition, these same viruses demonstrate a KgL-specific signal at ~15 kDa, which represents the predicted size of an unmodified form of KSHV gL that is devoid of signal peptide sequence. Interestingly, another ~22 kDa form of KSHV gL is also detected, though only in purified RRV KgH/KgL and KSHV virions, suggesting that modification and incorporation of this form of KSHV gL only occurs in viruses that also co-express KSHV gH. In addition, an ~18 kDa form of KSHV gL is detected strictly in RRV KgH/KgL virions, which may represent a full-length version of KSHV gL that has retained the signal peptide sequence and lacks detectable modifications (Fig. 2B). Furthermore, no KSHV gL-specific signal is detected in WT RRV BAC or RRV gLns virions, validating the specificity of the antibody. As equivalent amounts of infectious virus were loaded in each well in these experiments, the detection of slightly higher levels of MCP in RRV gLns, RRV KgL, and RRV KgH/KgL, relative to WT RRV BAC, may indicate the presence of more defective particles in these virus preparations.

**Fig 3:**
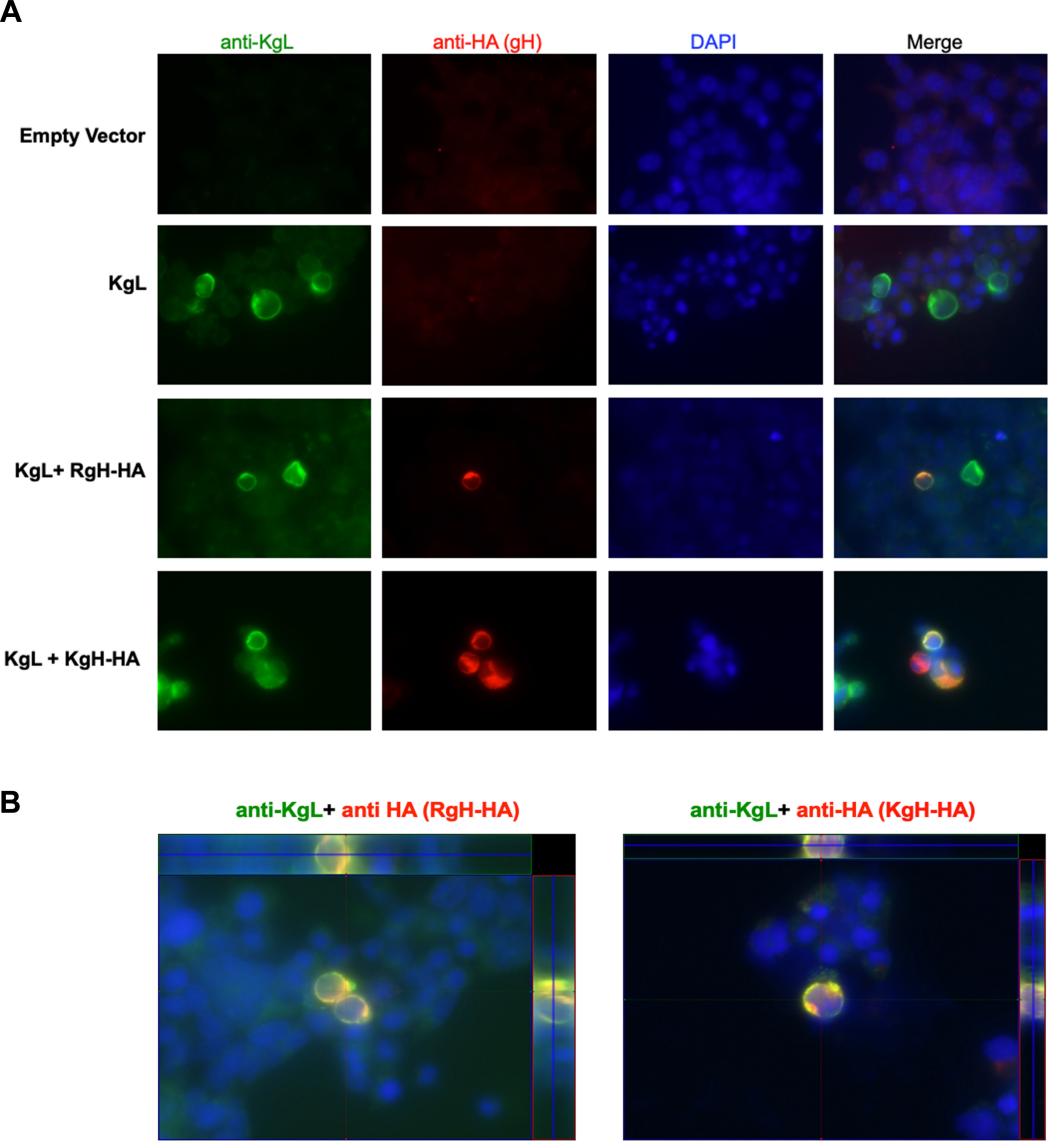
Immunofluorescence analysis to assess KSHV gL localization with RRV gH and KSHV gH in cells. HEK293T/17 cells were transfected with a vector expressing KSHV gL alone or in conjunction with vectors expressing C-terminal HA-tagged versions of RRV gH (RgH-HA) or KSHV gH (KgH-HA). Empty vector served as a negative control. Cells were fixed and stained with rabbit anti-KSHV gL antibody UK170 and mouse anti-HA antibody, followed by anti-rabbit-FITC and anti-mouse Texas Red secondary antibodies. DAPI staining was performed to visualize nuclei. (**A**) KSHV gL displays expression throughout the cytoplasm and at the plasma membrane and co-localizes with signal specific for RRV gH-HA and KSHV gH-HA, as indicated by yellow/orange staining patterns in merged images. (**B**) Z-stack images of stained cells further demonstrate the co-localization of KSHV gL with both RRV gH-HA and KSHV gH-HA in multiple planes in cells expressing these proteins. Side panels indicate the horizontal (top) and vertical (right side) cross-section of each image.

The ability of KSHV gL to be successfully incorporated into RRV KgL virions implies that this protein can interact and localize with RRV gH in infected cells, despite the inherent sequence variation between RRV gH and KSHV gH molecules. To assess whether KSHV gL and RRV gH co-localize within cells in which these proteins are expressed, immunofluorescence analysis was performed. For these experiments, 293T/17 cells were transfected with plasmids expressing KSHV gL and either RRV gH-HA or KSHV gH-HA, or an empty vector as a control. All samples were also co-transfected with RRV ORF57, which promotes the expression of RRV gH (42). After transfection and incubation, cells were fixed and stained with anti-KSHV gL or anti-HA antibodies and visualized by microscopy. These images demonstrate that KSHV gL, RRV gH, and KSHV gH are expressed in transfected cells, displaying extranuclear patterns of staining (Fig. 3A). Importantly, in cells co-expressing KSHV gL and either RRV gH-HA or KSHV gH-HA, signals for KSHV gL largely overlap with those of both forms of gH, demonstrating that KSHV gL, which lacks a transmembrane sequence, is capable of associating with RRV gH and KSHV gH in these cells. This is further demonstrated by z-stack analysis of co-transfected cells, in which signals for KSHV gL and either RRV gH or KSHV gH are detectable as co-localized signals in multiple planes (Fig. 3B). Importantly, these data demonstrate that KSHV gL can co-localize with RRV gH in cells, which would be necessary for the incorporation of KSHV gL into RRV KgL virions.

### RRV glycoprotein mutant and chimeric viruses display differing plaque morphologies and replication kinetics

**Fig 4:**
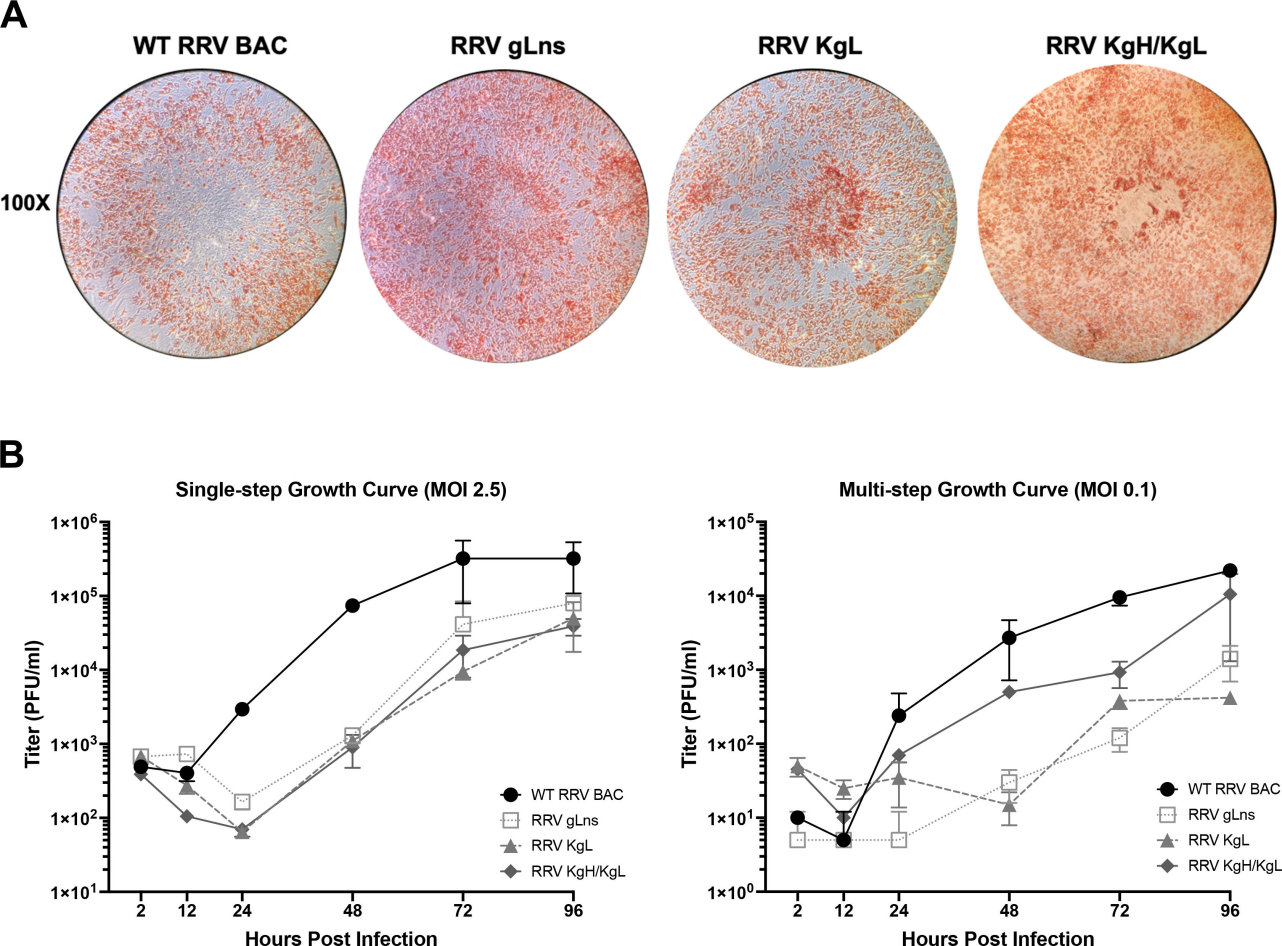
Plaque morphology and growth analysis of glycoprotein mutant and chimeric viruses. (**A**) 1^o^RF in 6-well dishes was infected with diluted stocks of WT RRV BAC, RRV gLns, RRV KgL, and RRV KgH/KgL incubated until the development of visible plaques and then fixed and stained with neutral red to visualize plaque morphology. Images depict a representative microscopic image of a single plaque from each well taken at 100× magnification. (**B**) Growth curve analysis of mutant and chimeric viruses in 1^o^RF. Cells were infected at an MOI of 2.5 (single step) or MOI of 0.1 (multi-step), and samples were harvested at the indicated time points. Plaque assay analysis was performed on 1^o^RF to determine viral titers in each sample. Error bars indicate standard deviation.

After the growth and purification of virus stocks, standard plaque assays were performed on 1^o^RF cells to directly compare WT RRV BAC, RRV gLns, RRV KgL, and RRV KgH/KgL. Based on general observations, it was initially noted that plaques produced by RRV gLns, RRV KgL, and RRV KgH/KgL display smaller sizes than those produced by WT RRV BAC. Staining of plaque assays coupled with microscopic examination of plaque morphology further revealed noticeable differences between viruses (Fig. 4A). Specifically, RRV gLns plaques demonstrate a smaller plaque size, while generally maintaining the appearance of WT RRV BAC plaques and possessing a clear center due to virus-associated cell death. However, plaques produced by RRV KgL display a different morphology overall, generating smaller plaques relative to WT RRV BAC, while also lacking a clear center that is seen with WT RRV BAC and RRV gLns infection and instead possessing the appearance of foci. Thus, both RRV gL and KSHV gL expression have effects on the morphology of RRV plaques, with KSHV gL altering the plaque phenotype of WT RRV BAC such that cells in the center of the plaque remain intact and do not demonstrate visible areas of cell death. When examining RRV KgH/KgL, it was evident that this virus also displays a unique plaque morphology, with generally larger-sized plaques relative to RRV gLns or RRV KgL, which lack clearly defined borders. Taken together, alterations in the expression of RRV gL and incorporation of KSHV gL and KSHV gH into RRV virions have observable effects on RRV plaque morphology, suggesting that variations in gp expression and incorporation may alter the ability of RRV to spread during infection, affect the ability of virus to kill infected cells, and induce variable effects on overall cellular morphology.

Growth kinetics were also assessed to determine the effects of RRV gL and KSHV gH/gL on lytic replication of chimeric viruses *in vitro* by performing standard growth curves in 1^o^RF. These results parallel general observations of the growth of these viruses in culture, with single-step and multi-step growth curves both indicating that RRV gLns, RRV KgL, and RRV KgH/KgL display decreased lytic replication relative to WT RRV BAC *in vitro* (Fig. 4B). In single-step growth curves, RRV gLns, RRV KgL, and RRV KgH/KgL demonstrate an approximate 1–2 log decrease in titers from 24 to 96 hours pi, suggesting that the absence of RRV gL negatively impacts the ability of RRV to infect and replicate even at a higher MOI, regardless of the expression of a homologous KSHV gL molecule or co-expression of KSHV gH and gL. Furthermore, a similar pattern of decreased replication in a multi-step growth curve suggests that viruses lacking RRV gL expression display decreased spread in culture relative to WT RRV BAC. However, co-expression of KSHV gH and KSHV gL in RRV KgH/KgL may slightly rescue this ability (Fig. 4B).

### Infection of B cells with RRV glycoprotein mutant and chimeric viruses

**Fig 5:**
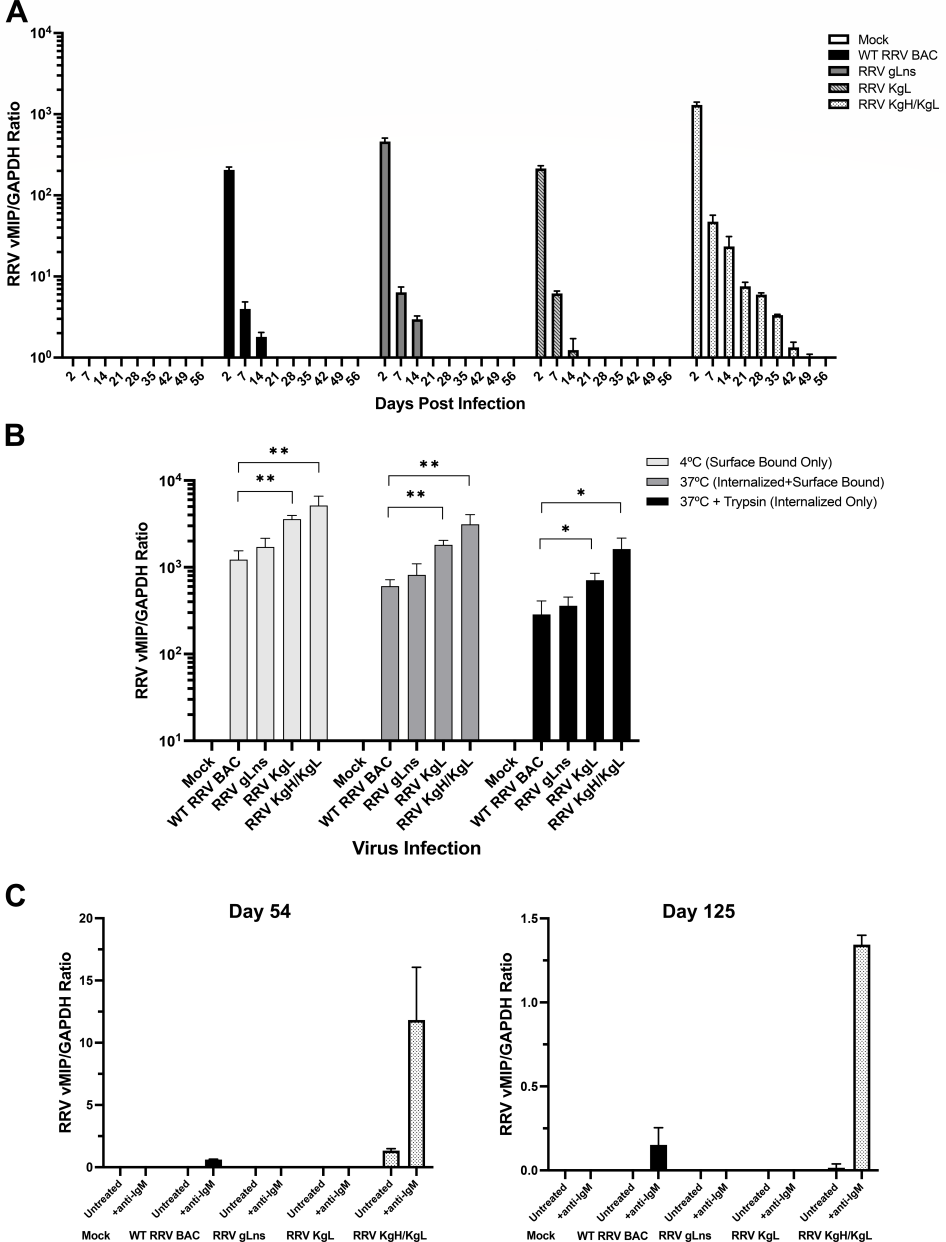
B cell infection and binding assays of RRV glycoprotein mutant and chimeric viruses. (**A**) Measurement of RRV genome copy levels during primary infection and establishment of latently infected BJAB cultures. BJAB cells were infected with WT RRV BAC, RRV gLns, RRV KgL, or RRV KgH/KgL at an MOI of 2, and DNA was isolated from cell samples at the indicated time points post-infection. qPCR was then performed using a primer and probe set specific for RRV viral macrophage inflammatory protein and normalized to GAPDH copy numbers to determine the relative amount of RRV genome copies in each sample (RRV vMIP/GAPDH ratio). Error bars indicate standard deviation. (**B**) BJAB cells were mixed with each virus at an MOI of 2 on ice, then incubated at 4°C for 2 hours to measure surface-bound virus, incubated at 37°C for 2 hours to measure internalized and any remaining surface-bound virus, or incubated at 37°C for 2 hours followed by treatment with trypsin to measure internalized virus only. After incubations, cells were washed with cold PBS, DNA was purified, and RRV vMIP/GAPDH ratios were determined by qPCR. Data represent the average of three independent experiments, and error bars indicate standard deviation. Relative to WT RRV BAC, RRV KgL and RRV KgH/KgL both display significantly higher levels of surface-bound virus (**P = 0.0011 and **P = 0.0097, respectively), internalized + surface-bound virus (**P = 0.0013 and **P = 0.0089, respectively), and internalized virus only (*P = 0.0176 and *P = 0.0138, respectively), as measured by unpaired *t* test. (**C**) Samples of latently infected BJAB cultures (from panel A) were taken at an early (day 54) and late (day 125) time point post-infection, treated with anti-human IgM to induce viral reactivation, or left untreated, and DNA was isolated for qPCR to determine RRV vMIP/GAPDH ratios in each sample. Error bars indicate standard deviation.

B cells are a critical natural target of KSHV and RRV infection and a site of viral latency *in vivo* (45), and KSHV-infected B cells can be directly involved in the development of viral-associated LPD (46). Therefore, to assess the ability of RRV gL mutant and RRV chimeras expressing KSHV gH and gL gps to infect B cells, we utilized human BJAB cells, a cell line that has been used as a model for B cell infection studies of both RRV and KSHV (47, 48) and in which RRV is capable of establishing a latent infection. Although RRV-infected BJAB cells do not produce readily quantifiable levels of lytic virus (48), viral genome copies can be used as a measure of infection. For these assays, BJAB cells were infected with WT RRV BAC, RRV gLns, and gp chimeric viruses at an MOI of 2, and DNA samples were isolated 2 days pi then weekly thereafter to allow for the measurement of viral infection levels by qPCR (Fig. 5A). These data demonstrate that all forms of RRV examined are capable of infecting B cells, with WT RRV BAC, RRV gLns, and RRV KgL achieving similar levels of infection, and RRV KgH/KgL displaying slightly higher levels of infection. The levels of infection detected for all viruses were highest at day 2 pi, with a gradual decline in detectable levels of viral genomes occurring through the initial period of infection and the ultimate establishment of a latent infection by day 21 in WT RRV BAC, RRV gLns, and RRV KgL-infected cells and day 56 in RRV KgH/KgL-infected cells. The ability of RRV gLns to infect B cells similarly to WT RRV BAC indicates that gL is not specifically required for RRV binding and entry in this cell type, while similar levels of infection by RRV KgL also suggest that the addition of KSHV gL to RRV does not dramatically affect the ability of RRV to infect B cells. However, infection with RRV KgH/KgL results in the detection of higher levels of virus relative to WT RRV BAC, RRV gLns, and RRV KgL at all time points up to day 42, suggesting that the presence of a KSHV gH/gL heterodimer may enhance the ability of RRV to establish an infection in B cells.

Although RRV KgH/KgL displays a higher level of infection in BJAB cells relative to WT RRV BAC, RRV gLns, and RRV KgL, it was uncertain whether this might be due to enhanced binding or an increased ability of this virus to fuse and enter cells. Therefore, to attempt to address this question, experiments to assess binding and entry were performed. For these assays, cells were infected at an MOI of 2.0 and then subjected to varying incubation conditions, including incubation at 4°C to measure levels of surface-bound virus, incubation at 37°C to measure surface-bound and internalized virus, and incubation at 37°C followed by trypsin treatment to allow the measurement of internalized virus only (49). After performing incubations and treatments, all cells were washed, DNA was isolated, and levels of viral genomes in each sample were measured by qPCR. These data demonstrate that RRV KgH/KgL and RRV KgL both bind to the surface of BJAB cells to significantly higher levels than WT RRV BAC, while RRV gLns binds similarly to WT RRV BAC (Fig. 5B). Interestingly, examination of virus internalization demonstrates that RRV KgL and RRV KgH/KgL are both capable of gaining significantly higher levels of entry into BJAB cells relative to WT RRV BAC, while RRV gLns displays infection levels equivalent to WT RRV BAC in both instances (Fig. 5B). Although an increase in initial cell surface binding of RRV KgH/KgL and RRV KgL may ultimately result in overall higher levels of virus internalization during infection, it remains possible that viral entry is also affected by the presence of KSHV gH and gL molecules in these virions. Regardless, these data suggest that the incorporation of a KSHV gH/gL or KSHV gL alone allows for the initial enhancement of RRV binding and entry into B cells immediately after exposure to virus, while at later time points pi, only RRV expressing KgH/KgL maintains elevated levels of infection relative to WT RRV BAC.

Despite the ability of all viruses examined to bind and enter B cells, it remained unknown whether these viruses all equally maintain a latent infection state in these cells or are capable of reactivation. Thus, latently infected BJAB cultures were stimulated with anti-IgM, a treatment that induces viral reactivation in this cell type (50). For these assays, cells were considered latently infected after day 49, a time point at which viral genome copy levels had stabilized to near background levels in all cultures (Fig. 5A). An early (day 54) and late (day 125) time point of each infected cell type were treated with anti-IgM. Results from this experiment indicate that RRV KgH/KgL is more capable of maintaining a long-term infection in BJAB cells compared to WT RRV BAC, which shows less reactivation at both time points. In addition, the absence of any detectable viral genome copies in RRV gLns or RRV KgL-infected BJAB cells after treatment with anti-IgM suggests a lessened ability of these viruses to establish a latent infection or maintain persistence in this cell type in culture (Fig. 5C). Therefore, RRV may require RRV gL in order to establish a more successful long-term infection in B cells, while replacement of RRV gH/gL with KSHV gH/gL promotes the ability of RRV to more efficiently infect and be maintained in this cell type. In general, these data demonstrate that chimeric forms of RRV expressing KSHV gL or KSHV gH/gL possess the ability to bind and infect B cells *in vitro*, suggesting these viruses are also likely capable of targeting and infecting this cell type *in vivo*.

### Infection of rhesus macaques with RRV glycoprotein mutant and chimeric viruses

**Fig 6:**
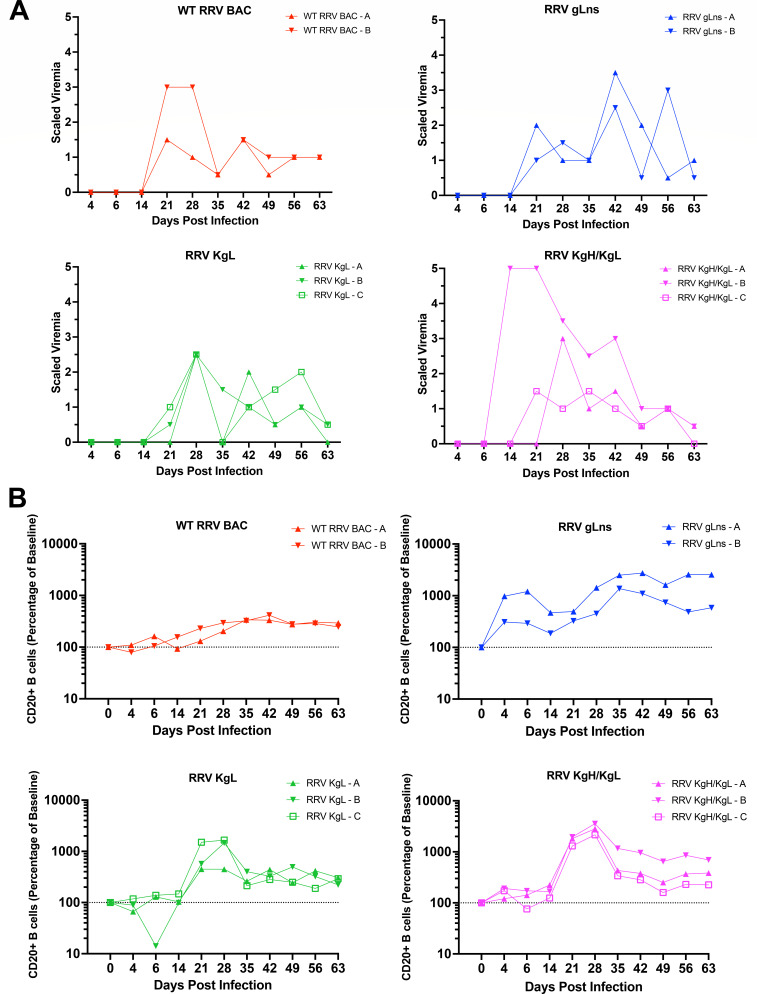
Development of viremia and B cell hyperplasia in infected rhesus macaques. (**A**) To assess the development of viremia during the acute phase of infection, PBMC samples were isolated from infected RM at the indicated time points, serially diluted 1:3, and co-cultured with primary rhesus fibroblasts. Viremia is defined as the limiting dilution factor where RRV CPE was detected, with a viremia scale of 1 representing the starting concentration of PBMCs (2 × 10^5^ PBMCs) utilized for co-culture. A viremia scale of 2 represents the first dilution where RRV CPE was detected in duplicate, and a maximum viremia scale of 5 indicates that all duplicate dilutions were positive for RRV CPE. A half scale indicates that RRV CPE was detected in one of the duplicates of the higher dilution. (**B**) The levels of total CD20 + B cells in infected RM were assessed by flow cytometry analysis of whole blood samples. All viruses examined induce an expansion of B cells in infected RM, indicating that RRV gLns, RRV KgL, and RRV KgH/KgL retain the ability to induce B cell hyperplasia, despite variances in gH and gL expression patterns from WT RRV BAC. Data are presented as percent change from baseline levels, which are set to 100% (dashed line).

A major impetus for the generation of RRV glycoprotein mutant and chimeric viruses is the development of these viruses as tools to examine the roles of RRV and KSHV gps in various aspects of infection, immunity, and pathogenesis *in vivo*, using an established NHP model system. Therefore, we initiated primary studies to assess the ability of RRV gLns, RRV KgL, and RRV KgH/KgL to infect RRV naive RM. For these studies, RM were infected with RRV gLns (two animals), RRV KgL (three animals), RRV KgH/KgL (three animals), or WT RRV BAC (two animals) as a control, with each animal receiving 5 × 10^6^ PFU of purified virus i.v. (Table 1). Samples were collected from each animal prior to infection (day 0), then at day 4, day 6, and weekly thereafter through day 63, a timeframe that encompasses the typical acute infection period for WT RRV in RM (16, 51). First, to determine whether RRV gp mutant and chimeric viruses are capable of establishing an acute infection in RM, viremia was measured via co-culture of purified PBMCs with 1^o^RF (18). Initial viremia was detected by days 21–28 pi in most animals, except for RRV KgH/KgL animal B, which demonstrated the earliest detectable viremia overall at day 14 pi and also displayed the highest level of viremia relative to all other animals (Fig. 6A). Although there was some variability, after the initial period of viremia, most animals resolved the infection by day 63 pi, displaying low to no detectable levels of virus by this time point. These data demonstrate that RRV gL is not required for RRV_17577_ infection of RM, which parallels observations made using a mutant form of RRV H26-95 lacking gL expression (38). Importantly, they also indicate that forms of RRV expressing KSHV gL or KSHV gH/gL are fully capable of infecting RM and establishing infection levels similar those observed with WT RRV BAC.

**TABLE 1:** Animals utilized in infection studies

Virus infection^*a*^	Animal ID	Sex^*b*^	Age^*c*^
WT RRV BAC	A	F	14
	B	F	8
RRV gLns	A	F	16
	B	M	13
RRV KgL	A	F	25
	B	M	15
	C	F	11
RRV KgH/KgL	A	F	12
	B	M	25
	C	F	16

^*a*^ Intravenous inoculation with 5 × 10^6^ PFU of BAC-derived RRV.

^*b*^ M, male; F, female.

^*c*^ Animal age in years.

An increase in total B cell numbers and the development of B cell hyperplasia are phenotypes typically associated with RRV infection of RM (12) and generally coincide with the onset of viremia in infected animals (16, 19). Examination of total CD20+ B cell numbers in animals infected with RRV gLns, RRV KgL, and RRV KgH/KgL indicates that all three viruses induce an expansion of B cell numbers after infection, similar to WT RRV BAC (Fig. 6B). The initial increase in CD20+ B cells occurs after 14 dpi in most animals, with the exception of RRV gLns-infected RM, which demonstrates a brief expansion starting from 4 to 6 dpi, before the onset of a period of secondary expansion. In addition, the peak levels of B cell expansion achieved in RRV gLns and gp chimeric virus-infected animals are higher relative to those infected with WT RRV BAC. However, due to animal-to-animal variation and small sample size of this cohort, the significance of these observations remains uncertain. Overall, however, all viruses induce an expansion in total CD20+ B cells after infection, with B cell numbers remaining elevated above baseline for the remainder of the infection, indicating that the absence of RRV gL expression or the replacement of RRV gL or gH/gL with their KSHV homologs may not have a major impact on B cell behavior *in vivo*.

**Fig 7:**
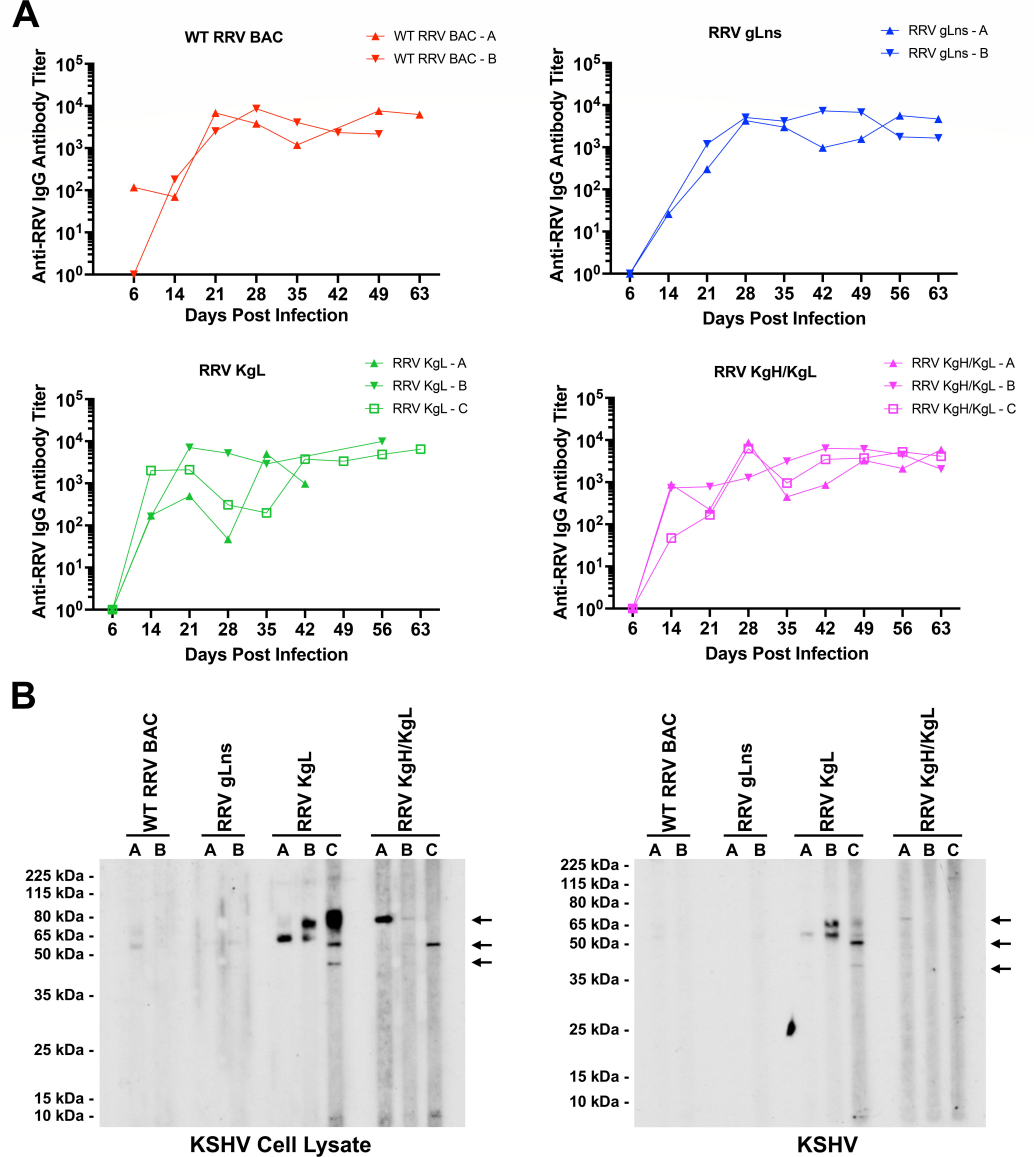
Development of antibody responses in infected rhesus macaques. (**A**) Plasma from infected RM was analyzed for the presence of RRV-specific antibodies via ELISA, demonstrating the development of measurable antibody responses against RRV in all infected animals. Measurements for some time points in specific animals are absent due to a lack of sufficient samples for use in this analysis. (**B**) To assess the presence of KSHV-specific antibodies in infected RM, day 49 pi, plasma samples from each animal were utilized in western blot analysis against lysates from induced iSLK.219 cells or purified KSHV. Each plasma sample was diluted 1:500, with the exception of samples from RRV KgL-C, RRV KgH/KgL-B, and RRV KgH/KgL-C, which were utilized at a dilution of 1:100. Signals that are specific to plasma samples from animals infected with RRV KgL or RRV KgH/KgL are noted with arrows.

To assess the ability of RRV glycoprotein mutant and chimeric viruses to induce immune responses against RRV, the levels of RRV-specific IgG in plasma from infected RM were assessed by ELISA. Despite some variability between animals and the absence of some time points for some animals due to sample availability, antibody responses against all viruses generally developed from 14 to 28 dpi, achieving peak levels from approximately 21 to 35 dpi, and remained elevated through the remainder of the acute infection period (Fig. 7A). Thus, forms of RRV lacking gL expression or expressing KSHV gL or KSHV gH/gL remain capable of inducing similar antibody responses as WT RRV BAC *in vivo*, demonstrating that these viruses are equally recognized by the immune system despite variations in gp expression profiles.

Given that RRV gp chimeras express KSHV antigens, the potential also exists for these viruses to induce the production of antibodies that are directed against KSHV. To determine the presence of KSHV-specific antibodies in infected RM, western blots were performed. For these assays, plasma samples from 49 dpi were diluted and used to probe KSHV-infected cell lysates or purified KSHV (Fig. 7B). Results from these experiments indicate that plasma from animals infected with RRV KgL or RRV KgH/KgL displays reactive signals against KSHV that are not detected in animals infected with WT RRV BAC or RRV gLns, demonstrating the presence of KSHV-specific antibodies only in animals infected with KSHV gp chimeric viruses. Specifically, using KSHV-infected cell lysates, plasma from two RRV KgL-infected and all three RRV KgH/KgL-infected RM detects specific signals at ~70 kDa (RRV KgL-B, RRV KgL-C, RRV KgH/KgL-A, and RRV KgH/KgL-B), ~50 kDa (RRV KgL-C and RRV KgH/KgL-C), and ~40 kDa (RRV KgL-C). Using purified KSHV, observed signals were lower in intensity, though KSHV-specific signals could still be detected in plasma samples from RRV KgL-B, RRV KgL-C, and RRV KgH/KgL-A. Interestingly, the sizes of the proteins detected in positive plasma samples from both RRV KgL-infected and RRV KgH/KgL-infected RM are similar, and no signals are detected at or above the predicted MW or the previously observed size of a modified form of KSHV gH (~80 and ~115 kDa, respectively) using plasma samples from any RRV KgH/KgL-infected RM. Taken together, this suggests that KSHV-specific antibodies generated in RM infected with RRV KgH/KgL are preferentially directed against KSHV gL, rather than KSHV gH. Furthermore, although RMs infected with forms of RRV expressing KSHV gL are capable of producing KSHV-specific antibodies, the observed sizes of the reactive proteins detected utilizing plasma samples range from ~40 to ~70 kDa (Fig. 7B), while the forms of KSHV gL that are detected when using a KgL-specific antibody in western blot analysis of purified RRV KgL, RRV KgH/KgL, or KSHV virions are smaller, ranging from ~15 to ~27 kDa (Fig. 2). This observation may indicate that antigen presentation during *in vivo* infection with RRV KgL and RRV KgH/KgL chimeric viruses results in the generation of antibodies that preferentially recognize other larger and more extensively modified forms of KSHV gL.

**Fig 8:**
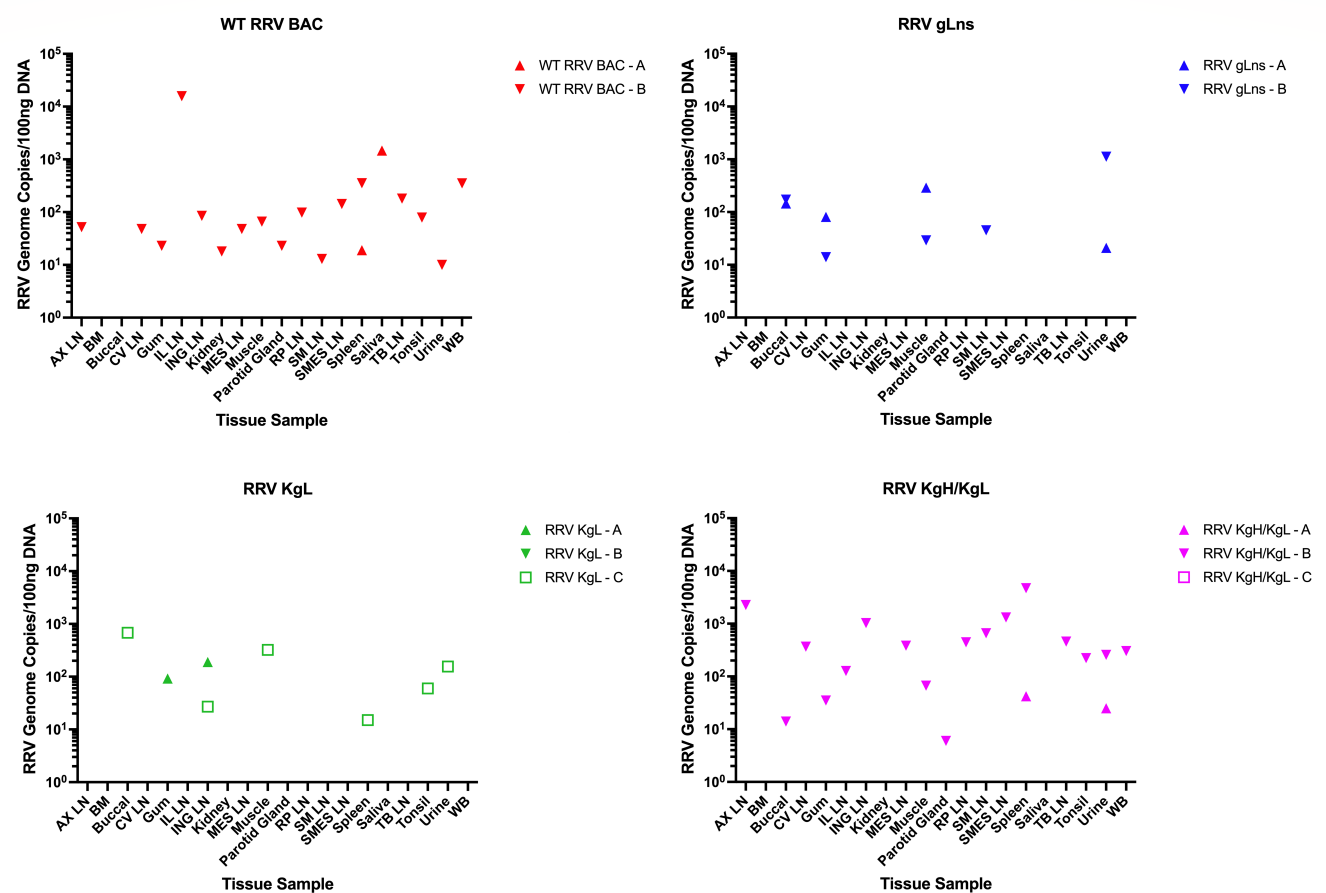
DNA viral loads in tissue samples of infected RM at the time of necropsy. Tissue samples, saliva, urine, and whole blood were collected from infected RM at the time of necropsy (WT RRV BAC-A d189 pi, WT RRV BAC-B d189 pi; RRV gLns-A d175 pi, RRV gLns-B d182 pi; RRV KgL-A d159 pi, RRV KgL-B d149 pi, RRV KgL-C d170 pi; RRV KgH/KgL-A d170 pi, RRV KgH/KgL-B d191 pi, RRV KgH/KgL-C d191 pi). DNA was isolated from each sample, and 100 ng was subjected to qPCR to assess the levels of RRV genomic DNA using a primer and probe set specific for RRV vMIP. Tissues analyzed include axillary lymph node (AX LN), bone marrow (BM), buccal tissue, cervical lymph node (CV LN), gum tissue, iliosacral lymph node (IL LN), inguinal lymph node (ING LN), kidney, mesenteric lymph node (MES LN), muscle, parotid gland, retropharyngeal lymph node (RP LN), submandibular lymph node (SM LN), superior mesenteric lymph node (SMES LN), spleen, saliva, tracheobronchial lymph node (TB LN), tonsil, urine, and whole blood (WB). Animals RRV KgL-B and RRV KgH/KgL-C displayed no detectable viral loads in any sample examined.

At the time of necropsy, a panel of tissue samples was collected from each animal, and DNA was isolated to assess the levels of viral DNA loads by qPCR (Fig. 8). Excluding RRV KgL-B and RRV KgH/KgL-C, all animals displayed detectable viral loads in at least two tissue samples, indicating that that RRV gLns, RRV, KgL, and RRV KgH/KgL are all capable of infecting a variety of tissues *in vivo*, similar to WT RRV BAC. In general, however, there was a large amount of variability in detectable viral loads both between animals and among different tissue samples, and no specific pattern was noted to directly indicate any alterations in tropism between the different viruses. Overall, these data suggest that gH and gL may not dramatically affect the tropism of RRV or KSHV *in vivo*.

## DISCUSSION

In our current study, we were able to successfully produce RRV expressing KSHV gL (RRV KgL), a double chimeric form of RRV expressing KSHV gH and gL (RRV KgH/KgL), and a mutant form of RRV lacking gL expression (RRV gLns). Although the growth properties of these viruses differed from that of WT RRV BAC, with all three displaying decreased lytic replication *in vitro* in 1^o^RF, high titer stocks of all viruses were successfully produced. However, despite our ability to generate a mutant RRV lacking gL expression, attempts at creating a form of RRV lacking gH expression were unsuccessful, as were attempts at creating a single chimeric form of RRV expressing KSHV gH. Specifically, RRV BAC clones containing mutations in ORF22 that prevent gH protein expression did not generate any apparent signs of virus production in 1^o^RF. These findings seem to parallel those performed with a mutant KSHV BAC lacking gH expression, which is capable of producing virus particles but displays an inability to replicate or be propagated in culture (43). Our inability to fully generate virus from a KSHV gH chimeric RRV BAC clone could suggest incompatibility of RRV gL with KSHV gH, and the possible lack of formation of functional KSHV gH/RRV gL dimers, or improper processing of KSHV gH in the absence of KSHV gL co-expression, both of which might limit the ability of this virus to infect and replicate in culture. Furthermore, as an RRV BAC clone lacking RRV gL expression is fully capable of producing virus that grows to high titers while only expressing RRV gH, it is plausible that KSHV gH lacks sufficient homology to fully replace RRV gH for the infection of target cells in the context of RRV or that RRV gL interacts with KSHV gH in RRV virions in a manner that blocks or interferes with the ability of this molecule to interact with target receptors to promote fusion.

Western blot analysis of chimeric viruses demonstrates that RRV KgH/KgL successfully incorporates KSHV gH into virions, while both RRV KgL and RRV KgH/KgL incorporate KSHV gL. However, variations in the number and size of KSHV gL-specific proteins detected between these viruses indicate only the common incorporation of a ~27 kDa modified form and a ~15 kDa unmodified form of KSHV gL into RRV KgL and RRV KgH/KgL virions, as well as KSHV virions, while the detection of a ~22 kDa modified form of KSHV gL strictly in RRV KgH/KgL and KSHV virions suggests that not all forms of KSHV gL are efficiently incorporated into RRV KgL virions. These observations are likely due to differences in the association of KSHV gL with RRV gH and KSHV gH, which is due to sequence variability between these molecules. Furthermore, the presence of an ~18 kDa form of KSHV gL strictly in RRV KgH/KgL virions may indicate incomplete processing of this molecule in the context of this virus, despite the presence of a functional KSHV gH molecule. Further work will be required to determine the specific pairing mechanisms of KSHV gL and RRV gH in the context of viral particle assembly and to determine if gp processing mechanisms might be altered in some instances due to expression in the context of a different viral background.

B cells represent a natural target of infection and latency for both RRV and KSHV *in vivo*, and importantly, our results indicate that RRV gp chimeric viruses expressing KSHV gL or KSHV gH/gL, as well as an RRV gLns, are all capable infecting this cell type *in vitro*. These findings are important as they indicate that these forms of RRV are also likely capable of infecting B cells *in vivo*. Interestingly, RRV expressing KSHV gH/gL appears to infect B cells to higher levels than WT RRV BAC, RRV gLns, or RRV KgL and demonstrates an increased ability to reactivate from these cells after the establishment of latency. In addition, increased levels of internalized RRV KgH/KgL and RRV KgL are detected early after infection of B cells, and although this could simply be attributable to an enhanced ability of these viruses to bind to the cell surface, it is also possible that effects of KSHV gL or KSHV gH/gL on post-binding steps could help promote virus fusion and entry in these cells; however, this remains unclear. In general, the overall enhanced infection levels observed with RRV KgH/KgL in BJAB cells may result from a better ability of the KSHV gH/gL heterodimer to interact with receptors expressed in a human-derived cell line that help promote virus binding, entry, and replication, though other factors could also contribute to this phenotype. Similarly, higher levels of reactivation observed in B cells latently infected with RRV KgH/KgL may indicate that this virus has a better ability to establish or maintain latency in infected B cells, which could be a direct result of higher initial levels of infection but could also result from the occurrence of more spontaneous reactivation and reinfection events in infected cultures that allow for enhanced persistence. Indeed, variations in receptor binding between gp mutant and chimeric viruses could play a major role in determining their ability to infect specific cell types. Assays to examine potential differences in Eph receptor binding between RRV glycoprotein chimeras in BJAB cells have been performed but have thus far been inconclusive and will continue to be the focus of future studies. Finally, it is important to note that B cell infection experiments were performed at equivalent MOI based on stock titers obtained via plaque assay on 1^o^RF, while binding and infection levels in these cells were measured by viral genome copy levels, which could have the potential to affect some of the observed differences in binding made in these experiments.

To assess the properties of RRV gp mutant and chimeric viruses *in vivo*, infection studies were performed in RM. Overall results from these studies indicate that forms of RRV lacking gL or expressing KSHV gL or KSHV gH/gL are equally capable of infecting RM, inducing B cell hyperplasia that is typically associated with RRV_17577_ infection and promoting the development of anti-RRV antibody responses and the production of antibodies that are reactive against KSHV. The ability of RRV gLns to successfully infect RM suggests that gL is not strictly required for RRV to establish an infection *in vivo*, which is in agreement with recent observations made by Hahn et al. (38) who utilized a form of RRV H26-95 lacking gL expression to infect RM. Our data extend these findings to demonstrate that gL is not required for the development of some RRV-associated pathologies in infected RM, as induction of B cell hyperplasia is not affected in the absence of gL expression. Furthermore, the ability of RRV KgL (paired with RRV gH) or RRV KgH/KgL (possessing a paired KSHV gH and KSHV gL heterodimer) to successfully infect and induce similar patterns of B cell expansion and antibody responses as WT RRV BAC in infected RM suggests that large variations in the sequences of gH and gL molecules expressed by RRV does not dramatically affect binding and entry mechanisms in target cells that are required for the establishment of infection *in vivo*. In general, our findings indicate that interactions of gH/gL complexes with receptors such as Ephs, which are considered critical for entry in some cell types *in vitro*, may not fully dictate whether RRV can successfully establish an infection *in vivo*. Indeed, given that RRV has been shown to utilize Eph-independent mechanisms to infect cell types such as fibroblasts *in vitro* (33), the ability of RRV to utilize non-gH/gL-dependent mechanisms or alternate entry receptors to gain entry to at least some cell types *in vivo* could be a factor. Future work will attempt to fully decipher the receptor usage patterns of RRV glycoprotein mutant and chimeric viruses both *in vitro* and *in vivo*.

Due to sampling limitations in this study, we did not have the ability to purify cell subsets from PBMC samples obtained from infected RM, and thus, we were unable to accurately assess infection levels within specific cell types in the peripheral blood of these animals, including B cells. However, due to the observed effects of all viruses on the development of B cell expansion, it is highly likely that RRV gLns, RRV KgL, and RRV KgH/KgL possess the ability to infect B cells *in vivo*. Furthermore, qPCR analysis of samples collected from each animal at the time of necropsy demonstrates that these viruses can all be detected in a variety of tissues *in vivo*, and although animal-to-animal variation likely has some effects on the overall levels of virus present in each sample, no differences were noted that indicate measurable changes in viral tropism of gp mutant or chimeric viruses, relative to infection with WT RRV BAC. Nevertheless, it will be of importance to further evaluate and define the specific tropism of these viruses in future *in vivo* studies in order to fully assess whether gH/gL molecules might affect the ability of RRV and KSHV to gain entry to certain tissue compartments.

The ability of all viruses examined to induce similar levels of RRV-specific IgG in infected RM demonstrates that gH and gL molecules are not strictly required for the development of immune responses against RRV after infection. Importantly, our data also reveal that RM infected with RRV chimeras expressing KSHV gL or KSHV gH/gL can develop antibodies that are reactive with KSHV. Overall, the induction of antibody responses against KSHV gH and gL in RM infected with RRV KgL or RRV KgH/KgL might be anticipated, particularly given that KSHV gH/gL has been shown to be the predominant antigenic determinant for neutralizing antibodies in humans infected with KSHV (40). Of note, however, is the fact that antibodies generated against KSHV in RRV KgH/KgL infected RM appear to preferentially target KSHV gL even in the presence of KSHV gH co-expression, which may indicate that KSHV gL is more immunogenic *in vivo*. One unexpected finding was the observation that the sizes of KSHV-specific proteins detected with plasma samples from RRV gp chimeric-infected RM are larger than the forms of KSHV gL we identified in virions of both purified RRV gp chimeras and KSHV. These data suggest that the antibodies produced during *de novo* infection of RM with RRV KgL or RRV KgH/KgL may recognize modified forms of KSHV gL that are not detected using a KSHV gL-specific antibody. Further studies will aim to better define the specificity of the antibodies produced by RRV gp chimeric viruses and the epitopes they may target.

Overall, the successful generation of chimeric forms of RRV_17577_ expressing KSHV envelope gps has many implications. Not only can these viruses be utilized *in vitro* to help determine whether RRV and KSHV envelope gps display functional overlap or possess varied functions in virus entry and infection but they can also be further developed as unique tools to help assess the contributions of individual viral envelope gps to various aspects of infection, immunity, and disease development *in vivo*. Ultimately, in conjunction with the RRV/RM infection model, RRV chimeras provide a novel system allowing for the assessment of the functionality of vaccine and treatment strategies that target envelope gps for the prevention of KSHV infection in humans.

## Data Availability

Complete genomic sequences are available in GenBank for RRV gLns (accession number PP101847), RRV KgL (accession number PP101848), and RRV KgH/KgL (accession number PP101849).

## SUPPLEMENTAL MATERIAL

Supplemental figures jvi.01923-24-s0001.docx Figures S1 to S3.

ASM does not own the copyrights to Supplemental Material that may be linked to, or accessed through, an article. The authors have granted ASM a non-exclusive, world-wide license to publish the Supplemental Material files. Please contact the corresponding author directly for reuse.
