# Supplementary material for: Generation of chimeric forms of rhesus macaque rhadinovirus expressing KSHV envelope glycoproteins gH and gL for utilization in an NHP model of infection
Source: J Virol. 2025 Jan 21;99(2):e01923-24. doi: 10.1128/jvi.01923-24 (PMC11852781; doi:10.1128/jvi.01923-24)
Supplement: Supplemental figures — Figures S1 to S3. [file jvi.01923-24-s0001.docx]

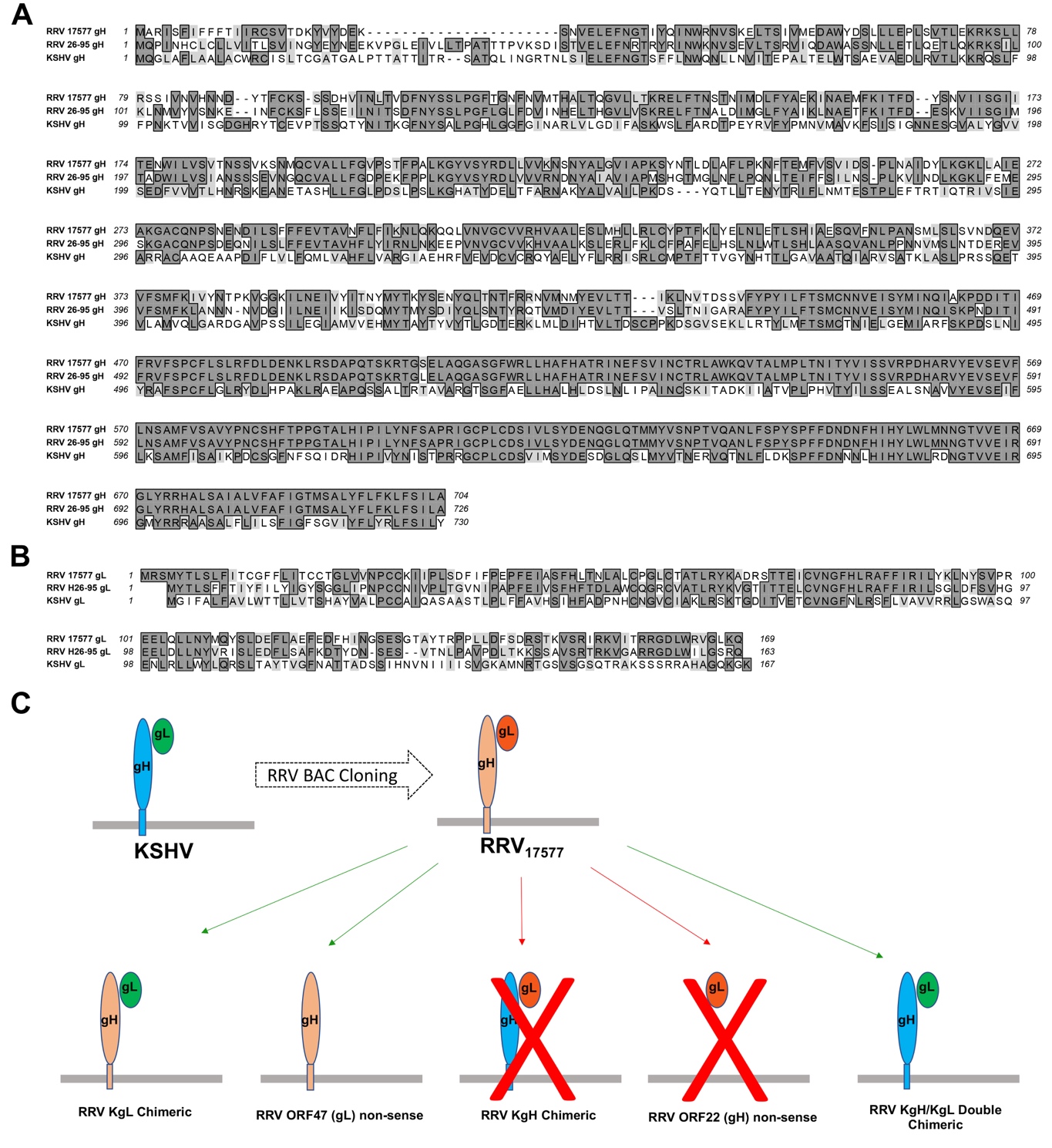


**Figure S1.** Comparison of RRV and KSHV gH and gL, and overview of glycoprotein mutant and chimeric viruses. Amino acid alignment of (A) gH and (B) gL from RRV and KSHV. Sequences used include RRV_17577_ gH (NP_570762.1) and gL (NP_570788.1), RRV H26-95 gH (AAF60000.1) and gL (AF210726.1), and KSHV BAC16 gH (QFU18817.1) and gL (QFU18842.1). Sequences were aligned using ClustalW. Dark gray shading indicates identity, and light gray shading indicates similarity. (C) Diagram of RRV glycoprotein mutant and KSHV glycoprotein chimeric viruses produced utilizing the RRV_17577_ BAC. Images with a red X indicate virus stocks that were unable to be generated from the RRV_17577_ BAC due to lack of sufficient virus production.


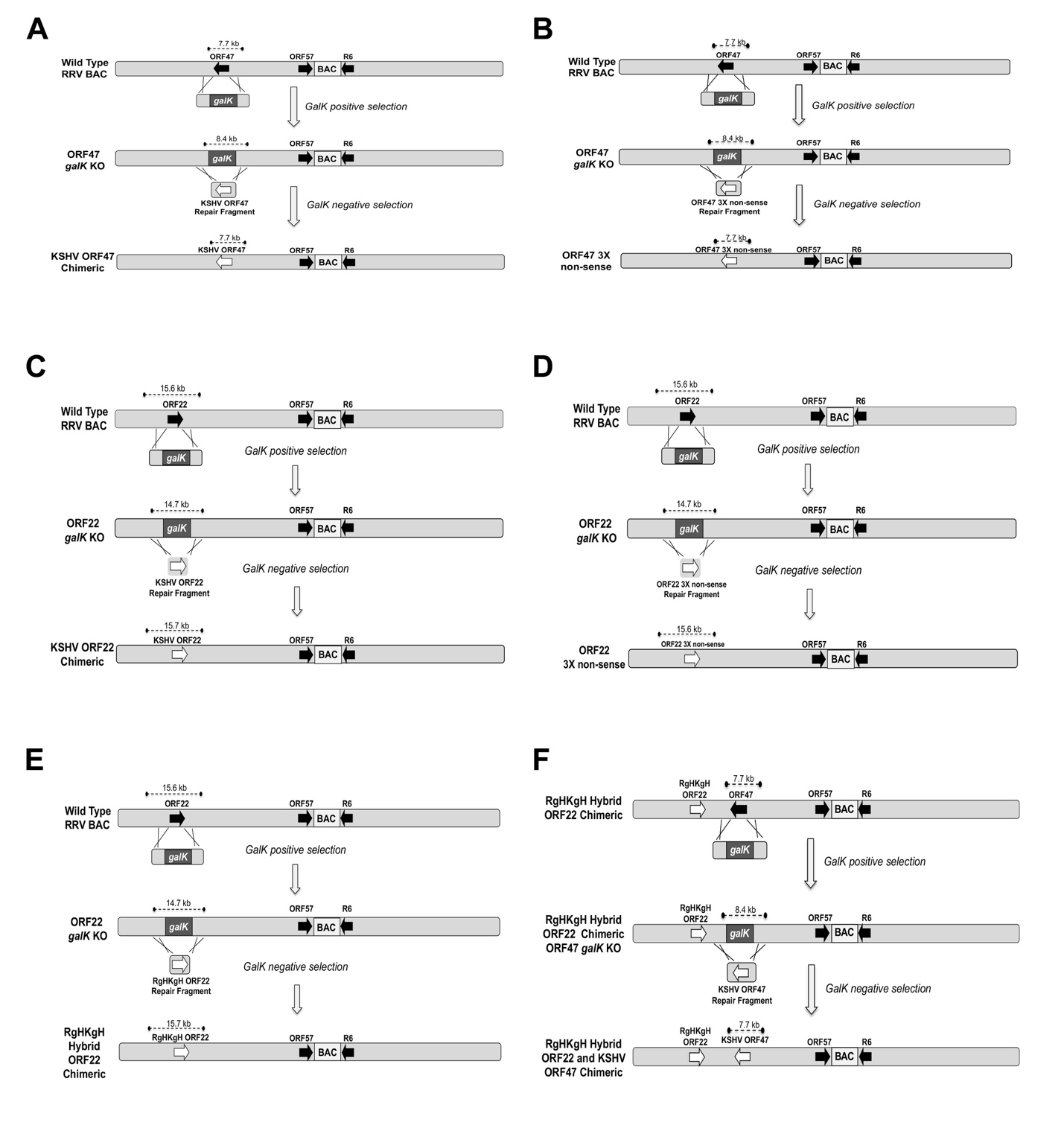


**Figure S2.** Cloning strategies utilized to generate glycoprotein mutant and KSHV glycoprotein chimeric RRV_17577_ BAC clones. (A) KSHV ORF47 (gL) chimeric BAC, (B) RRV ORF47(gL) 3X non-sense mutant BAC, (C) KSHV ORF22 (gH) chimeric BAC, (D), RRV ORF22(gH) 3X non-sense mutant BAC, (E) RgHKgH ORF22 hybrid chimeric BAC, and (F) RgHKgH ORF22 hybrid/KSHV ORF47(gL) double chimeric BAC. Dashed lines indicate the BamHI restriction digestion fragments encompassing region of *galK* insertion and repair for each clone, with sizes of each fragment noted.

**
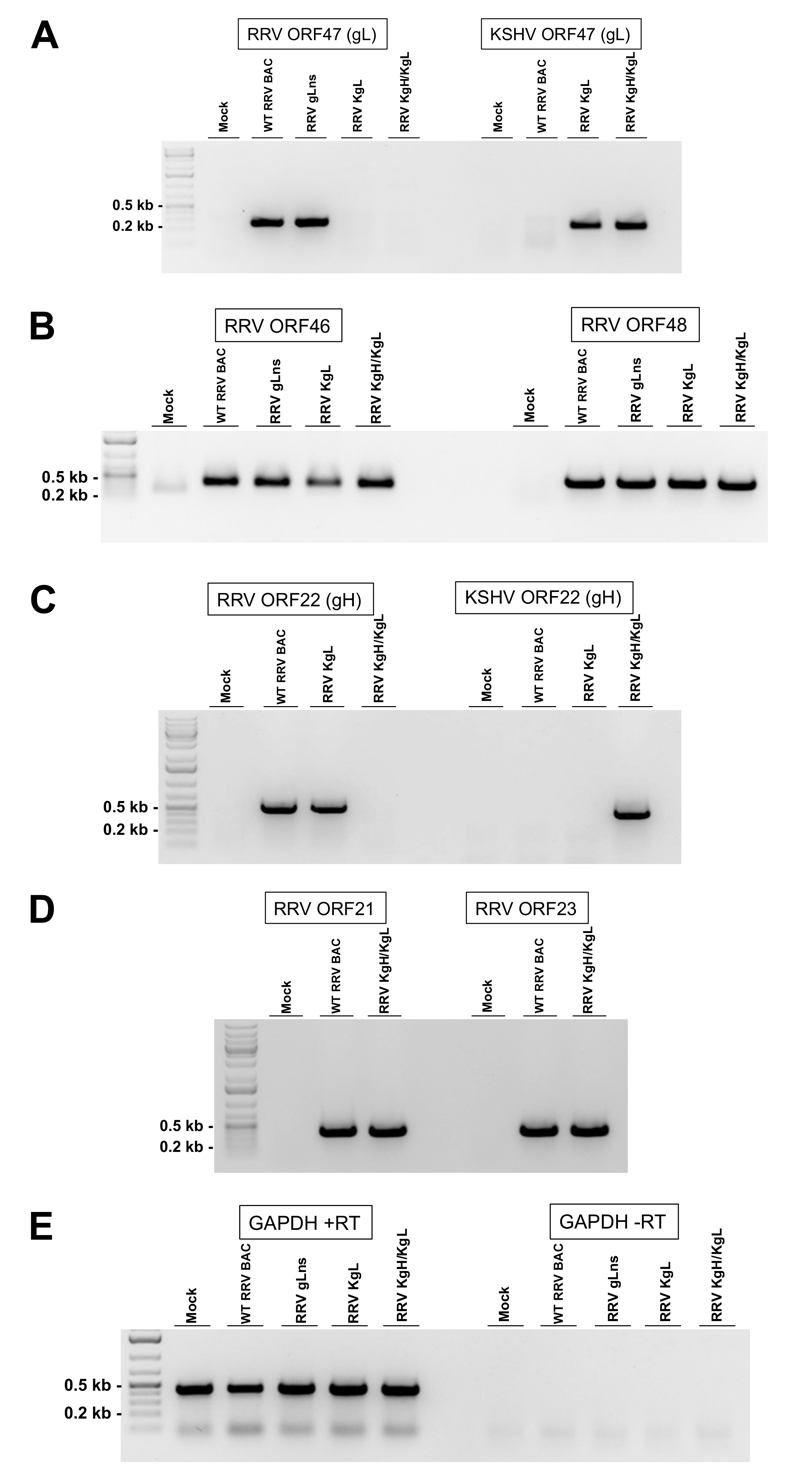
**

**Figure S3.** RT-PCR analysis of repair insert gene expression in glycoprotein mutant and chimeric forms of RRV. 1^o^RF were infected with WT RRV BAC, RRV gLns, RRV KgL, or RRV KgH/KgL, at an MOI of 2, and RNA was harvested at 72 hrs post infection for RT-PCR analysis using primers specific for (A) RRV ORF47(gL) and KSHV ORF47(gL), (B) RRV ORF47 neighboring genes ORF46 and ORF48, (C) RRV ORF22 (gH) or KSHV ORF22(gH), and (D) RRV ORF22 neighboring genes ORF21 and ORF23. (E) Reactions performed using GAPDH primers with or without RT demonstrate the absence of DNA contamination in all RNA samples used in this assay. RT-PCR performed using primers specific for RRV ORF47 demonstrates that RRV gLns, which contains point mutations in the 5’ region of ORF47, expresses RRV ORF47 transcripts to similar levels as WT RRV BAC, while RRV KgL and KgH/KgL lack any detectable RRV ORF47 transcripts. In the case of RRV KgL and KgH/KgL chimeric viruses, both viruses express KSHV ORF47 transcripts to similar levels. Analysis of RRV ORF46 and ORF48 expression further indicates that insertion of stop mutations in ORF47 sequence of RRV gLns, or replacement of RRV ORF47 with KSHV ORF47 sequence in RRV KgL and RRV KgH/KgL, does not alter the transcription of either flanking gene. Analysis of ORF22 indicates similar transcription levels of RRV ORF22 in RRV KgL and WT RRV BAC, the complete absence of RRV ORF22 transcripts in RRV KgH/KgL, and the expression of KSHV ORF22-specific transcripts in RRV KgH/KgL, in which hybrid RRV/KSHV ORF22 sequence is inserted. Finally, analysis of ORF21 and ORF23 transcript levels in RRV KgH/KgL indicate that transcription of neither flanking gene is altered due to the insertion of KSHV ORF22 sequence into the RRV genome.
